# Supplementary material for: ASCL1 induces neurogenesis in human Müller glia
Source: Stem Cell Reports. 2023 Nov 30;18(12):2400–17. doi: 10.1016/j.stemcr.2023.10.021 (PMC10724232; doi:10.1016/j.stemcr.2023.10.021)
Supplement: Document S1. Figures S1–S6 and Table S1 [file mmc1.pdf]

Stem Cell Reports, Volume *18*

Supplemental Information

ASCL1 induces neurogenesis in human Müller glia

Juliette Wohlschlegel, Connor Finkbeiner, Dawn Hoffer, Faith Kierney, Aric Prieve, Alexandria D. Murry, Alexandra K. Haugan, Isabel Ortuño-Lizarán, Fred Rieke, Sam A. Golden, and Thomas A. Reh

Figure legends

Figure S1

Markers of the presumptive fovea are already present by D57 (FWK8)

(A) Top: 2D sub-stack of a D57 fetal eye immunolabeled with OTX2 (green) and Recoverin (RCVRN, magenta). Bottom: IF whole-mount of the eye shown from the posterior. Grid boxes show the dimensions of the intact volume. Arrow points out the optic nerve; arrowhead shows the presumptive fovea. (B) D57 fetal retinal section immunolabelled with VSX2 (red), OTX2 (green), RCVRN (magenta) and DAPI (grey). Scale bar = 100 µm. Abbreviations: FWK: Fetal week; PF: presumptive fovea; ON: optic nerve; T: temporal; N: nasal.

Figure S2

SnRNA-seq and SnATAC-seq analysis of the D59 sample alone.

(A) UMAP plot of the D59 (FWK8) sample colored by cell type clusters. (A') UMAP with the different phases of the cell cycle, showing that MG are mostly in G1**.** Abbreviations: MPC: multipotent progenitor cells, Npre: neurogenic precursors cells, RGC: retinal ganglion cells, CON: cone photoreceptors, BIP: bipolar cells, MG: muller Glia, AST: astrocytes, HOR: horizontal cells, cyNpre: neurogenic precursors cells in the mitotic cycle. (B) Dot plot showing the expression of cell-type specific genes in the different cell clusters. (C) Feature plots showing the relative expression of glial markers RLBP1, SLC1A3, and NFIA. (D) Scatterplot of multipotent progenitors (MPCs) vs Muller glia (MG): genes that are significantly different in expression are colored and the top 10 differentially expressed genes are labeled. RLBP1 is significantly more highly expressed in the MG than in the MPCs, but it is not in the top 10 genes**.** (E) Scatterplot of enriched transcription factor binding motifs in peaks enriched in either MPCs or MGs as a percentage of cells. Transcription factor motifs that correspond to relative differences in RNA expression (shown in D) are labeled in panel E.

Figure S3

SnRNA-seq and SnATAC-seq analysis of the merged dataset (D59, D76C, and D76P).

(A) Dot plot showing the gene expression in the different cell clusters. (B) Stacked Bar plot showing the percentages of each cell cluster split by age (D59, D76C, and D76P). (C) Top GO biological process analysis for the MG (red) and MPC (blue) clusters for cells that are in the G1 phase of the cell cycle. (D) Scatterplot of accessible chromatin peaks that contain significantly higher abundance peaks specific to MG (red) or specific to MPC (blue).

Figure S4

PAX2+ cells in retinal organoids and retinospheres are likely astrocytes.

(A) FD150 (FWK21) fetal retinal section immunolabelled with RLBP1(red), VSX2 (green), PAX2 (cyan) and DAPI (grey). ONL: outer nuclear layer; INL: inner nuclear layer; GCL: ganglion cell layer. Scale bar = 50 µm. (B) PAX2+ are mostly absent in retinal organoids, PAX6 (red), SOX2 (green), PAX2 (cyan) and DAPI (grey). Scale bar = 100 µm. Arrowheads show PAX2+ cells. (C) MG cultures derived from RO and passaged once immunolabelled with the same markers as in (A) and (B). Scale bar = 100 µm. (D) PAX2+ cells are present in some retinospheres and (E) are more abundant in MG cultures (arrows) derived from fetal retinal tissues (retinospheres). Scale bar = 100 µm.

Figure S5

Both MG derived from human fetal retina and from retinal organoids can be reprogrammed into neurogenic precursors *in vitro.*

(A) Heatmap showing the expression the top 25 marker genes expressed in the neuronal cluster after *ASCL1* expression (from Fig 6E) averaged and normalized across the neuronal clusters of the Mutiome data (from Fig 2) and (B) plotted onto the merged UMAP of the Multiome data (from Fig 2B). (C) Pseudotime values on a UMAP plot of snRNA-seq results from MG cultures derived from retinal organoids that were infected with *ASCL1* expressing virus. (D) Stacked Bar plot showing cluster composition for the two different conditions: CTL and ASCL1. (C) and (D) are further analyzed in Figure5. (E) Pseudotime values on a UMAP plot of scRNA-seq results from MG cultures derived from fetal retina retinospheres that were infected with ASCL1 expressing virus. (F) Stacked Bar plot showing cluster composition for the two different conditions: CTL and ASCL1. (E) and (F) are further analyzed in Figure 6. (G) Integrated UMAP plot of the two different datasets (Fig 5 and Fig 6), split by conditions (G') MG cultures derived from retinospheres (Fig 6), (G'') MG cultures derived from retinal organoids (Fig 5).

Figure S6

ASCL1 overexpression combined with Notch signaling inhibition induces MG-derived OTX2+ neurons.

(A) Feature plots showing the expression values of HES1, ID1, ID3, (top panel) in the dataset obtained from retinal organoids (Fig 5), (lower panel) in the dataset obtained from fetal retina (retinospheres) (Fig 6). (B) Schematic of the experimental timeline with the Notch inhibitor. (C) Merged UMAP of the MG cultures (CTL: no virus, ASCL1 only and ASCL1 combined with Notch

inhibitor). (D) Feature plots showing the expression of neurogenic precursor maker (HES6) and neuronal markers (ELAVL3, DCX and RBFOX3). (E) Merged UMAP splits by condition; red (CTL), green (ASCL1 only) and blue (ASCL1 overexpression combined with Notch signaling inhibition). (F) Stacked Bar plots showing neurogenic and neuron cluster composition for the ASCL1 alone and the ASCL1 + Notch inhibitor conditions. (G) Feature plot showing OTX2 expression on the merged UMAP split by conditions. Arrow indicates the OTX2 neuronal cluster present only in the ASCL1+ Notch inhibition signaling condition.

Video 1

The presumptive fovea is detectable as early as D59 in the human fetal retina.

A 3D rendering of a D59 (FWK8) human fetal eye after whole mount immunostaining and clearing. Recoverin (RCVRN, magenta) labelling is only restricted to one specific region of the retina, temporal to the optic nerve. The green channel (IBA1) was used to visualize the structure retina.

Antibodies	Source (brand)	Identifier (catalogue identifier)	Dilution
Mouse anti-CRALBP (RLBP1)	abcam	ab15051	1/200
Rabbit anti-GFAP	DAKO	Z0334	1/500
Goat anti-SOX2	SC-17320	SC-17320	1/300
Rabbit anti-SOX9	Millipore	Ab5535	1/300
Sheep anti-CHX10	EXALPHA	X1179P	1/300
Mouse anti-PAX6	DSHB	Pax6-s	1/50
Rabbit anti-PAX2	Biolegend	901001	1/100
Goat anto-OTX2	R&D Systems	BAF1979	1/300
Mouse anti-TUJ1	Biolegend	802001	1/300
Rabbit anti-RCVRN	Chemicon	AB5585	1/1000
Rabbit anti-PH3	NOVUS Biologicals	NB600-1168	1/300
Mouse anti-HUCD	Life Technologies	A-21271	1/200
Goat anti-DCX	Santa Cruz	D3015	1/300
Guinea pig anti-IBA1	Synaptic Systems	234-004	1/1000
Mouse anti-Ki67	Leica	NCL-L-ki67-MM1	1/300
Mouse anti-CalBindin	Millipore	AB1778	1/300

Donkey anti-goat 568	Invitrogen	A11057	1/300
Donkey anti-goat 488	Jackson	705-545-147	1/300
Donkey anti-mouse 488	Jackson	715-605-150	1/300
Donkey anti-mouse 568	Invitrogen	A10037	1/300
Donkey anti-mouse 647	Invitrogen	A32787	1/300
Donkey anti-rabbit 488	Invitrogen	A21206	1/300
Donkey anti-rabbit 568	Invitrogen	A10042	1/300
Donkey anti-rabbit 647	Invitrogen	A31573	1/300
Donkey anti-sheep 488	Invitrogen	A11015	1/300

Table 1. List of antibodies. Columns present information about the antibodies, source, identifier, and dilution used for immunofluorescence or whole mount.

Experimental procedure

Organoid cultures

The human pluripotent stem cells line H7- BRN3-td Tomato was maintained as colonies on Matrigel coated well plates supplemented with StemFlex medium (Thermo Fisher Scientific). Medium was changed every other day. Reaching confluency, stem cells were passaged for maintenance using ReLeSR (StemCell Technologies) following manufacturer's instructions. Cells were then split, with the ratio depending on the confluency of the cells, once a week. For the differentiation protocol, we used Dispase (Gibco) (1mg/ml) to lift the colonies and let them self-aggregate into embryoid bodies (EBs) in T25 flaks with a medium containing 1/1 StemFlex and Neural induction medium (NIM, DMEMf12, 1% N2 supplement, 1% MEM non-essential amino acids, 1% Pen-Strep). Two days later, EB medium was completely switched to NIM medium. On day 6, BMP4 (R&D systems) was added to the flask and, on day 7 EBs were plated on regular cell culture treated plates with 10% FBS. Medium was changed the next day (NIM). On day 20 plated EBs were lifted using P1000 tips and were then transferred into low adhesion plates supplemented with retinal differentiation medium (RDM: DMEM/DMEMf12, 1% MEM non-essential amino acids, 2% B27, 5% FBS, 1% Pen-Strep). Retinal organoids were maintained for long term *in vitro* culture with medium changing 3 times a week on Monday, Wednesday, and Friday. Since, retinal organoids have distinct features which enable selection, non-retinal forebrain cells were manually removed. All cells (stem cells, EBs and retinal organoids) were maintained in an incubator at 37°C with 5% $CO2$.

Freezing protocol

After removing the medium, plated cells were harvested using Accutase (Millipore Sigma) for 5-8 mins, 37°C. The cell suspension was then spun down at 300 RPM for 7 min at 4°C. Supernatant was removed, and cells were resuspended using a medium containing 50 % of neuronal medium, 40% FBS and 10% DMSO. Cells were then preserved in a cryotube at -80°C.

Notch inhibition in MG cultures

Four days after *ASCL1* overexpression with the lentiviral construct, we used 10 µM of PF-03084014-hydrobromide (PF4014) (Millipore Sigma) to inhibit Notch signaling *in vitro*. Medium was not changed during the three days following Notch inhibition.

Single-cell multiome sequencing data preparation and analysis

The Cellranger ARC pipeline (2.0) was used to demultiplex samples and align reads to the GRCh38 genome. First, we used 'cellranger-arc mkfastq' to convert BCL files to fastq and demultiplex samples. This was done independently for the ATAC and RNA reads. Next, cellranger arc count was used to combine ATAC and RNA reads from each cell (using cell barcodes and samples of origin), map Tn5 insertions to the genome, and align RNA reads to the genome. Data was further processed in R version 4.2.2 (R core team, 2021).

Data from cellranger arc count were loaded into Signac (1.9.0) (Stuart et al., 2021) /Seurat (4.3.0)(Hao et al., 2021). The RNA assay was processed using the default Seurat pipeline. Reads were normalized using 'NormalizeData', variable features were Identified using 'FindVariableFeatures', gene expression across cells was scaled using 'ScaleData', and PCs were created using RunPCA(). Additionally, all samples used the RNA data for clustering and UMAP. This was done using Seurat's RunUMAP() function (to create the UMAP), and the 'FindNeighbors' and 'FindClusters' functions to find clusters in the neighbor space. Clusters were identified via differential gene expression and expression of known marker genes. Cell cycle phase was approximated using Seurat's 'CellCycleScoring' function which uses a curated list of genes to determine if cells are in the S, G2M, or G2, G1 phase of the cell cycle.

The ATAC assay for each object was created using the barcoded fragment files from Cellranger arc count. Signac's 'CallPeaks' function was used to call peaks. This function calls Macs2 (2.2.7) (Zhang et al., 2008) on the fragment files to identify peaks in the sample. Cell fragment

pairs were mapped to the Macs2 called peaks using Signac's 'FeatureMatrix' function which returned a peak by cell matrix. This matrix was used for all downstream analysis. Nucleosome signal strength and TSS enrichment for each cell were calculated using Signac's 'NucleosomeSignal' and 'TSSEnrichment' functions respectively. Outliers in the QC metric categories were removed as per Signac's standard processing guidelines.

Peaks were annotated with motifs for transcription factor binding sites in the Jaspar 2020 database using Signac's 'AddMotifs' function. Signac's 'RunChromvar' function was then run to find motif accessibility z-scores across cells. The z-score values were used in the motif heatmaps and featureplots. Coverage plots were made using Signac's 'CoveragePlot' function.

Monocle 3

Pseudotime was calculated using Monocle 3. The Seurat object was loaded into Monocle3 using SeuratWrapper's 'as.cell_data_set()' function. Clusters were rerun using Monocle3's 'cluster_cells()' function and the lineage graph was created using Monocle3's 'learn_graph()' function. Monocle3's 'order_cells()' function was used to assign the pseudotime root using known markers of precursor cell types. For the D59 sample there was a small bipolar branch that was visible on UMAP but did not cluster out from the rest of the OTX2 lineage. To isolate this group of cells we used Monocle3's 'choose_graph_segments()' to select cells on this branch segment.

Cascade Heatmap plots

Cascade plots were generated as previously described (Finkbeiner et al., 2022; Todd et al., 2022). To create the heatmap, we used chromvar activity scores of the top variable motifs across the different lineages arranges over pseudotime. Similarly, RNA heatmaps were generated using the z-score (scale data) gene expression values corresponding to each of the variable motifs in the chomvar heatmap, following the same order over pseudotime.

Combining datasets

Before combining datasets, we first ran all previous computational steps on each sample independently. If the samples we were combining contained accessibility assays we merged peaks from all samples that were to be combined using BEDOPS (-m)(Neph et al., 2012) on the Macs2 peaks for each sample. Signac's 'FeatureMatrix' function was then run with the resulting merged peaks to create a new peak by cell matrix. All assays in the samples that were to be

combined were then downsampled to similar average read depth using R and DropletUtils ‘downsampleMatrix’ function. If the samples were sequenced at the same time samples were merged using Signac/Seurat’s ‘merge’ function, and standard Seurat/Signac normalization, dimensional reduction, clustering, and visualization was performed as described above. For integrated samples (Reprogramming 2 and integrated reprogramming 2 and 4) the objects were split by samples then integrated with Seurat’s ‘IntegrateData’ function using anchors calculated using Seurat’s ‘FindIntegrationAnchors’ function. Then normalization, dimensional reduction, and visualization steps were repeated as previously described.

Peak-gene and TF-gene linkage

For Multiome, samples genes were linked to peaks using Signac’s ‘LinkPeaks’ function using the default parameters. To investigate whether targets of a specific transcription factor were up or downregulated between groups, differential gene expression was performed between the groups of interest and differentially expressed genes were aggregated by group. For each differentially expressed gene linked peaks with a positive score were selected giving us peaks linked to differentially expressed genes for each population. Percentage of genes with TF binding motifs in linked peaks for DE genes for each population were calculated by inputting the linked peaks from each population into Signac’s ‘FindMotifs’ function. The resulting percentages were plotted against each other as a scatterplot colored according to the average $\log_2$ fold change from Seurat’s ‘FindMarkers()’ function.

References

Finkbeiner, C., Ortuño-Lizarán, I., Sridhar, A., Hooper, M., Petter, S., & Reh, T. A. (2022). Single-cell ATAC-seq of fetal human retina and stem-cell-derived retinal organoids shows changing chromatin landscapes during cell fate acquisition. *Cell Reports*, *38*(4). https://doi.org/10.1016/j.celrep.2021.110294

Hao, Y., Hao, S., Andersen-Nissen, E., Mauck, W. M., Zheng, S., Butler, A., Lee, M. J., Wilk, A. J., Darby, C., Zager, M., Hoffman, P., Stoeckius, M., Papalexi, E., Mimitou, E. P., Jain, J., Srivastava, A., Stuart, T., Fleming, L. M., Yeung, B., … Satija, R. (2021). Integrated analysis of multimodal single-cell data. *Cell*, *184*(13), 3573-3587.e29. https://doi.org/10.1016/j.cell.2021.04.048

Neph, S., Kuehn, M. S., Reynolds, A. P., Haugen, E., Thurman, R. E., Johnson, A. K., Rynes, E., Maurano, M. T., Vierstra, J., Thomas, S., Sandstrom, R., Humbert, R., & Stamatoyannopoulos, J. A. (2012). BEDOPS: High-performance genomic feature operations. *Bioinformatics*, *28*(14), 1919–1920. https://doi.org/10.1093/bioinformatics/bts277

Stuart, T., Srivastava, A., Madad, S., Lareau, C. A., & Satija, R. (2021). Single-cell chromatin state analysis with Signac. *Nature Methods*, *18*(11), 1333–1341. https://doi.org/10.1038/s41592-021-01282-5

Todd, L., Jenkins, W., Finkbeiner, C., Hooper, M. J., Donaldson, P. C., Pavlou, M., Wohlschlegel, J., Ingram, N., Rieke, F., & Reh, T. A. (2022). Reprogramming Müller glia to regenerate ganglion-like cells in adult mouse retina with developmental transcription factors. *SCIENCE ADVANCES*. https://doi.org/10.1126/sciadv.abq7219

Zhang, Y., Liu, T., Meyer, C. A., Eeckhoute, J., Johnson, D. S., Bernstein, B. E., Nussbaum, C., Myers, R. M., Brown, M., Li, W., & Shirley, X. S. (2008). Model-based analysis of ChIP-Seq (MACS). *Genome Biology*, *9*(9). https://doi.org/10.1186/gb-2008-9-9-r137
