# Supplementary material for: ASCL1 induces neurogenesis in human Müller glia
Source: Stem Cell Reports. 2023 Nov 30;18(12):2400–17. doi: 10.1016/j.stemcr.2023.10.021 (PMC10724232; doi:10.1016/j.stemcr.2023.10.021)
Supplement: Document S2. Article plus supplemental information [file mmc3.pdf]

Stem Cell Reports
Article
OPEN ACCESS

ASCL1 induces neurogenesis in human Müller glia

Juliette Wohlschlegel,[1] Connor Finkbeiner,[1] Dawn Hoffer,[1] Faith Kierney,[1] Aric Prieve,[1] Alexandria D. Murry,[1] Alexandra K. Haugan,[1] Isabel Ortuño-Lizarán,[1] Fred Rieke,[2] Sam A. Golden,[1,3] and Thomas A. Reh[1,4,*]

[1]Department of Biological Structure, University of Washington, Seattle, WA, USA
[2]Department of Physiology and Biophysics, University of Washington, Seattle, WA, USA
[3]Center of Excellence in Neurobiology of Addiction, Pain, and Emotion (NAPE), University of Washington, Seattle, WA, USA
[4]Institute for Stem Cells and Regenerative Medicine, University of Washington, Seattle, WA, USA
*Correspondence: tomreh@uw.edu
https://doi.org/10.1016/j.stemcr.2023.10.021

SUMMARY

In mammals, loss of retinal cells due to disease or trauma is an irreversible process that can lead to blindness. Interestingly, regeneration of retinal neurons is a well established process in some non-mammalian vertebrates and is driven by the Müller glia (MG), which are able to re-enter the cell cycle and reprogram into neurogenic progenitors upon retinal injury or disease. Progress has been made to restore this mechanism in mammals to promote retinal regeneration: MG can be stimulated to generate new neurons *in vivo* in the adult mouse retina after the over-expression of the pro-neural transcription factor *Ascl1*. In this study, we applied the same strategy to reprogram human MG derived from fetal retina and retinal organoids into neurons. Combining single cell RNA sequencing, single cell ATAC sequencing, immunofluorescence, and electrophysiology we demonstrate that human MG can be reprogrammed into neurogenic cells *in vitro*.

INTRODUCTION

Diseases that lead to degeneration of retinal cells are among the leading causes of blindness worldwide (Stein et al., 2021). Although regeneration of retinal cells is a robust process in some non-mammalian vertebrates (Todd and Reh, 2022), this process does not occur in mammals. After injury in several species, including zebrafish, the Müller glia (MG) re-enter the cell cycle and generate cells with characteristics of retinal progenitors. The resulting progenitors proliferate and generate new neurons (Goldman, 2014; Wan and Goldman, 2016). By contrast, mammalian MG respond to damage by activating a reactive process associated with inflammation called gliosis (Bringmann et al., 2009; Dyer and Cepko, 2000).

The molecular mechanisms involved in retinal regeneration have been well studied in fish, amphibians, and birds, and several key factors are critical for neural regeneration from MG (Todd and Reh, 2022). One of these factors, the pro-neural transcription factor (TF) *Ascl1*, is expressed after injury in fish and birds, but not mammals (Fausett et al., 2008; Fisher and Reh, 2001). Furthermore, *Ascl1* (*Ascl1a*) is required to initiate neurogenesis from MG in fish (Fausett et al., 2008). When *Ascl1* is overexpressed in mouse MG, the cells acquire a progenitor-like phenotype after injury, similar to that of the injured fish retina. These MG-derived progenitor-like cells generate new neurons (Jorstad et al., 2017; Pollak et al., 2013; Ueki et al., 2015), which are functional and form connections with existing neurons (Jorstad et al., 2017; 2020). *Ascl1*-reprogrammed MG generate progenitors that primarily differentiate into bipolar or amacrine-like cell types (Jorstad et al., 2017; 2020). However, when additional TFs, *Atoh1*, or the combination of *Pou4f2* and *Islet1*, are co-expressed with *Ascl1*, the reprogrammed MG generate neurons that resemble retinal ganglions cells (RGCs), demonstrating that additional TFs can control the fates of the *Ascl1*-reprogrammed MG (Todd et al., 2021; 2022).

These results support the concept that MG might serve as a source of retinal repair in human retinal diseases; however, we do not know whether the same factors will induce neurogenesis from human MG (Salman et al., 2021). Indeed, we know very little about the factors that normally regulate the development of the human retina, although many of the same mouse developmental genes are present in human retina, gene regulatory networks differ (Eldred et al., 2018; Lu et al., 2020; Lyu et al., 2021). Additionally, the human retina has some characteristics the mouse retina lacks, such as the fovea, and it is possible that foveal MG may differ in their ability to be reprogrammed to a neurogenic state (Reichenbach and Bringmann, 2020).

We, therefore, undertook a study of human MG and tested their ability to be reprogrammed to a neurogenic state. In this study, we demonstrate that (1) human MG arise and differentiate sooner than previously demonstrated, in a region corresponding to the presumptive fovea; (2) using two different models, we can generate dissociated cultures of fetal human MG; (3) *ASCL1* expression in dissociated MG cultures induces a neurogenic program in the human MG; (4) *ASCL1* remodels the chromatin and induces a neurogenic progenitor state by activating retinal progenitor genes; and (5) the MG-derived progenitor-like cells generate new neurons, based on their morphology, gene expression, and electrophysiological

This is an open access article under the CC BY license (http://creativecommons.org/licenses/by/4.0/).

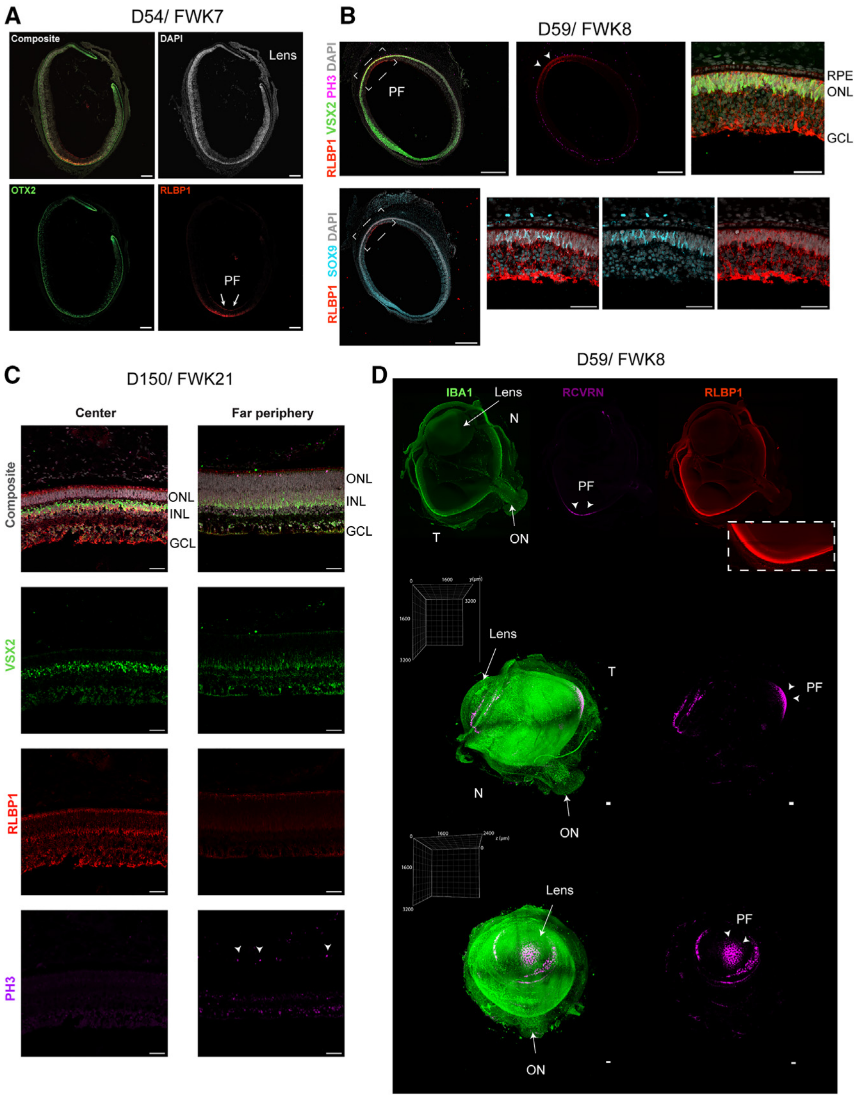

Figure 1. MG appear around fetal week 8 in the PF of the human fetal retina

(A–C) Immunostaining of MG in the developing human retina at (A) D54/FWK7, (B) D59/FWK8, and (C) D150/FW21. (A) MG and RPE are labeled with RLBP1 (red); RPE, bipolar, and photoreceptors cells are labeled with OTX2 (green), and DAPI (gray). Scale bar, 200 μm.

(B) (Top) MG are co-stained with RLBP1 (red) and VSX2 (green) in a region (white arrowheads) that does not contain PH3+ cells (magenta). (Bottom) MG are co-stained with RLBP1 (red) and SOX9 (cyan). DAPI (gray). Scale bar, 500 μm in low-magnification images. Scale bar, 50 μm in higher magnification images.

(C) MG are present in the central retina at 150 days, but not in the far periphery. RLBP1 (red) and VSX2 (green), DAPI (gray). Progenitor cells are labeled with PH3 (magenta). Scale bar, 50 μm.

(D) Volumetric imaging of a D59 human fetal eye after whole mount staining and clearing. (Top) A 2D sub-stack (20/1,370) with maximum intensity projection of the fetal eye immunolabeled with IBA1 (green), RCVRN (magenta), and RLBP1 (red). Inset shows some RLBP1+ cells in the RPE and in the retina. (Bottom) IF whole-mount of the fetal eye shown from side and front orientations. Grid boxes show dimensions of the intact volume. Arrowheads show the PF. Scale bar, 100 μm. GCL, ganglion cell layer; INL, inner nuclear layer; N, nasal; ON, optic nerve; ONL, outer nuclear layer; T, temporal.

properties. These results provide evidence of the potential regenerative capacity of human MG.

RESULTS

Characterization of MG development in the human fetal retina

To derive human MG from either fetal retina or retinal organoids, we first needed to better characterize their development. There is currently not much known about MG differentiation in humans, due to a lack of markers to discriminate them from the retinal multipotent progenitor cells (MPC) and limited access to human tissues at late stages of gestation (Hoang et al., 2020; Hu et al., 2019; Lu et al., 2020; Reichenbach and Bringmann, 2020; Tworig and Feller, 2022). Moreover, the development of the retina is not a homogeneous process, and the temporal central retina, including the future fovea develops and matures more than one month earlier than the periphery (Hendrickson, 2016; Hendrickson et al., 2012; Hendrickson and Yuodelis, 1984; Hoshino et al., 2017).

To define when the MG first arise during human retinal development, we used a combination of immunofluorescence (IF) on sections and cleared intact human fetal retinas. Previous studies reported the presence of MG at 77 days (fetal week [FWK] 11) and later in the human central retina (Hoshino et al., 2017; Lu et al., 2020; Sridhar et al., 2020). By contrast, we find IF labeling for the MG expressed protein, RLBP1, as early as 54 days (FWKs 7–8) of gestation in the central retina, in a region of temporal retina that presages the future fovea (Figure 1A). Contrary

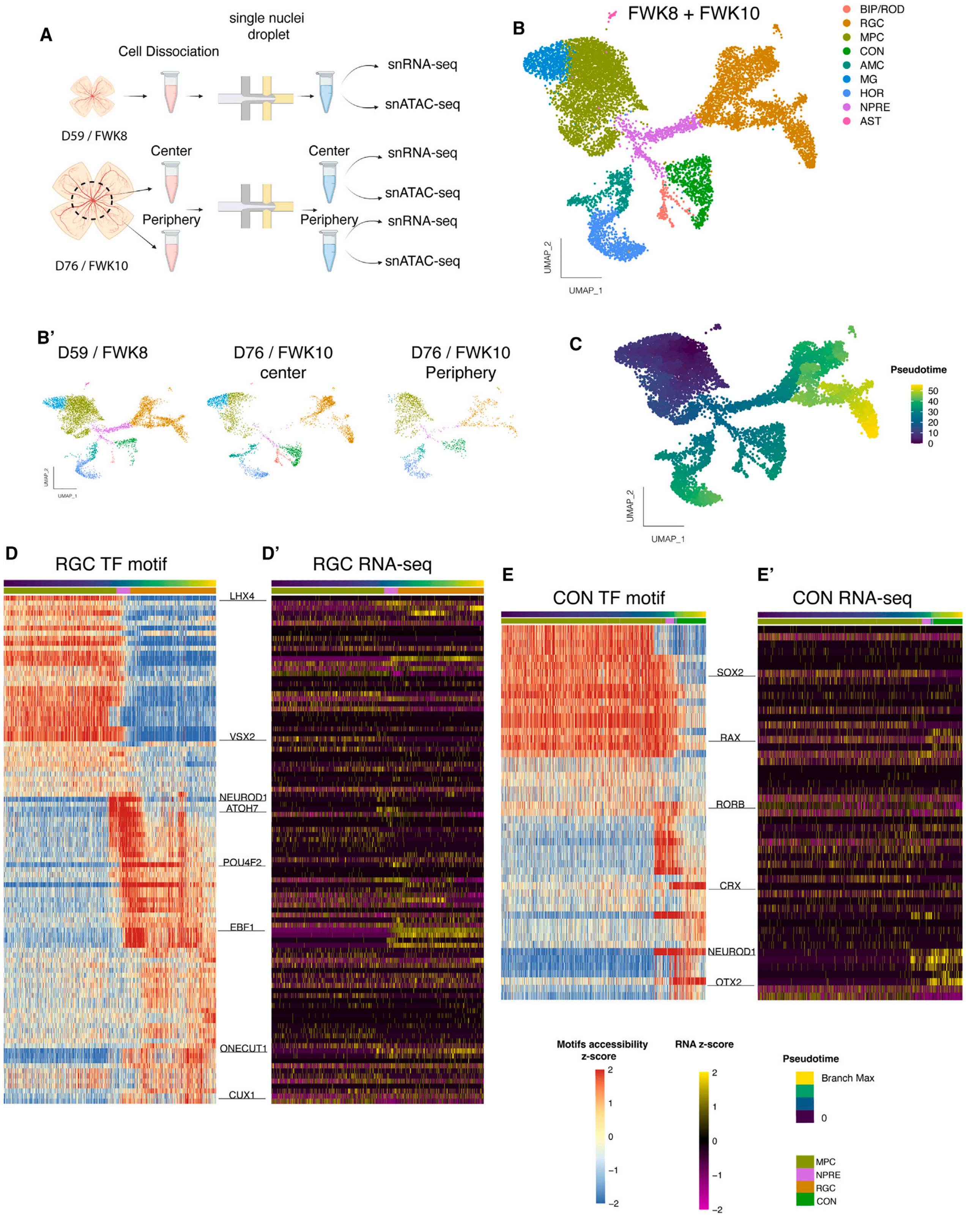

(legend on next page)

to other MG markers, RLBP1 is specific to MG (and the retinal pigment epithelium [RPE]) and, to the best of our knowledge is not present in the MPC (Sridhar et al., 2020). At 59 days (FWK8), we confirmed the presence of MG with additional IF markers, VSX2 and SOX9, along with RLBP1 (Figure 1B). Birth dating studies in other species have shown the MG are among the last cell types to be generated by the MPC; as a result, the appearance of RLBP1 in the presumptive fovea at fetal day 59 (FD59) correlates with the loss of markers of mitotic proliferation, such as PH3 (Figure 1B, arrowheads) (Cepko et al., 1996; Hoshino et al., 2017). In more mature retinal samples, MG differentiation then spreads from the central temporal retina to more peripheral regions. MG cells can be identified in the temporal periphery at approximately FD150 (FWK 21); however, they are still absent from the far periphery (Figure 1C). Although some MPC remain in the far peripheral retina at FD150, a large part of the retina contains MG at this stage (Figure 1C).

Early foveal development can be visualized within intact human fetal eye samples using whole-mount tissue clearing and IF staining protocols paired with volumetric three-dimensional (3D) light sheet fluorescent microscopy (LSFM) imaging (Vigouroux et al., 2020) (Figure 1D and Video S1). This approach conserves spatial relationships and provides an intact view of IF localization across the whole eye, without *a priori* selection of regions of interest, like in standard section-based IF. Intact D59 human fetal eye was immunostained for IBA1 (microglia), Recoverin (RCVRN; photoreceptors and bipolar cells) and RLBP1 expression (Figure 1D, top). IBA1 IF shows microglial cells present throughout the retina, while RCVRN expression is confined to the presumptive fovea (PF), temporal to the optic nerve. As noted above in sections, RLBP1 is also observed within the PF (Figure 1D, top inset), although this is difficult to cleanly visualize due to RLBP1 labeling within the RPE. We used 3D reconstruction (Figure 1D, bottom) to visualize RCVRN (magenta) localization across the intact eye (green), characterizing the spatial distribution of RCVRN labeling throughout the intact PF. Parallel analysis of a younger sample (D57) recapitulated the same expression pattern of RCVRN in the temporal side of the retina at the PF (Figures S1A and S1B).

Previous studies have described the transcriptome and accessible chromatin of the developing human retina but have not specifically focused on MG development. Since our IF evidence indicated that MG were already present in the PF as early as FWK8, we collected additional samples of fetal retina and processed them for combined single nuclei ATAC sequencing (snATAC-seq) and single nuclear RNA sequencing (snRNA-seq) (Multiome) to characterize the first MG in human retina. We collected two different fetal retinal samples, a day 59 (FWK 8) and a day 76 (FWK 10); for the second sample, we dissected the central retina, including the PF (76C) from the peripheral regions (76P) and processed the two different regions separately (Figure 2A). For the day 59 sample, data from 5,652 nuclei were analyzed using Seurat (Figure S2A). For the day 76 sample, data from 3,178 nuclei for the central retina and 1,739 nuclei for the peripheral retina were analyzed. To identify the MG in the single nuclei data, we merged the three datasets together and generated a single UMAP plot (Figure 2B). To determine the cell types present in the different clusters in the UMAP plot, we used known marker genes, such as *POU4F2* (RGCs), *OTX2* (photoreceptors and bipolar cells), *PRDM1* (photoreceptors) and *PTF1A* (amacrine cells) (Figures 2B, S2B, and S3A). In Figure 2B′, the integrated UMAP, split by sample, shows the presence of the different cell types over time and per region, confirming earlier reports . Cell clusters from the day 59 and day 76C largely overlap; however, at the later staged of the central retina, there were fewer cells composing the MPC and neurogenic precursor (Npre) clusters, and more cells present in late-generated neuronal clusters (amacrine, rod, and bipolar cells). Interestingly, the day 76P sample is still mostly composed of undifferentiated cell types and early generated neuronal clusters (RGCs, cones, and horizontal cells) and thus seems to be less advanced compared with the other samples (Figures 2B′ and S3B). Cells were next arranged in pseudotime, with the beginning of the branch in the MPC cluster and the tip of the branch ending in the desired neuronal cluster (Figure 2C). We next analyzed the different TF motifs associated with the accessible chromatin over pseudotime and generated "cascade plots" motifs in addition to the corresponding RNA expression for the RGC (Figures 2D and 2D′) and cone (Figures 2E and

Figure 2. SnRNA-seq and SnATAC-seq of the developing human retina
(A) Schematic of the single cell Multiome experiment.
(B) UMAP plot from the snRNA-seq merged datasets (FWK8 + FWK10) colored by cell type. AMC, amacrine cells; AST, astrocytes; BIP/ROD, bipolar and rod photoreceptor cells; CON, cone photoreceptors; HOR, horizontal cells; MPC, multipotent progenitor cells; Npre, neurogenic precursors; MG, muller glia; RGC, retinal ganglion cells. (B′) Distribution of the cells projected onto UMAP plots and split by conditions.
(C) Pseudotime values for FWK8 + FWK10 cells.
(D) Heatmap showing the cascade of TF motif accessibility variation found in the RGC lineage over pseudotime (D′) and the corresponding RNA expression for each TF in the RGC lineage.
(E and E′) Similar to (D and D′), respectively, but for the cone lineage.

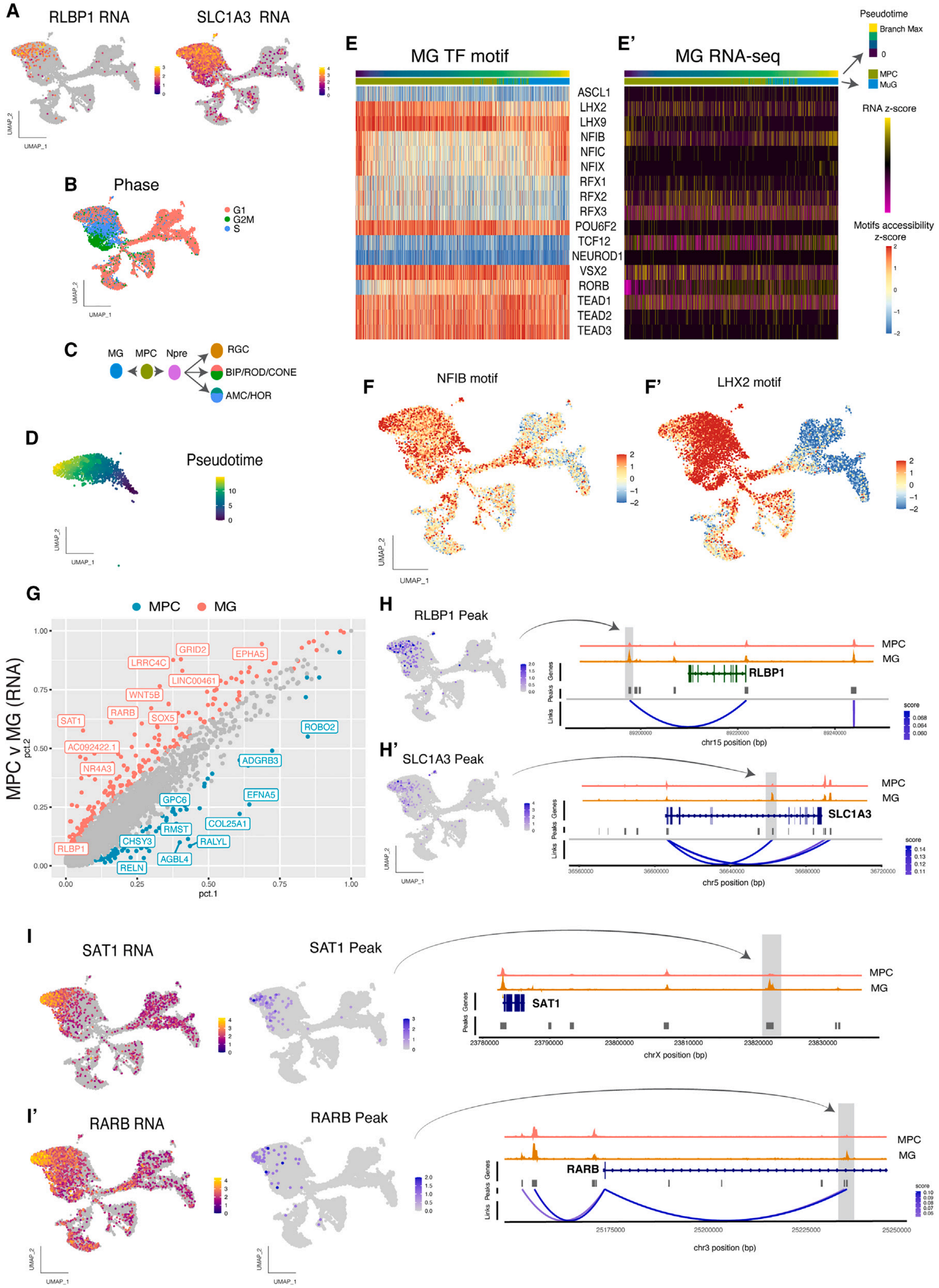

(legend on next page)

2E′) clusters. Cascade plots of enriched motifs for each cluster (RGC and cone) are consistent with previous reports showing a downregulation of progenitor motifs (VSX2, SOX2) followed by a progressive increase in neurogenic motifs (ASCL1, ATOH7, and NEUROD1) and cell-type-specific neuronal motifs (POU4F2 for RGCs and OTX2/CRX for cones) (Finkbeiner et al., 2022; Lyu et al., 2021).

As our focus is the development of the MG in the human retina, we next used our Multiome data to find one cluster that could be identified as MG by the expression of established markers: RLBP1 and SCL1A3 (Figures 3A and S2C). Although the MG are quite similar to the MPC, the MG express genes typical of the G1 phase of the cell cycle, while the MPC express S and G2 phase mitotic cell cycle genes (Figures 3B and S2A′). As we, and others, have previously noted, MG seem to differentiate from the MPC without entering the Npre stage (characterized by *ATOH7* expression), unlike the neuronal cell types, which pass through this stage on their way to terminal differentiation (Figure 3C) (Finkbeiner et al., 2022; Lyu et al., 2021). Similarly, to the RGC and Cone clusters, we also generated a cascade plot for motifs enriched in accessible DNA in the MG cluster. Cells were ordered in pseudotime from MPC to MG, keeping only the cells in G1 to minimize the effect of the cell-cycle genes (Figure 3D). Although, at this age, the MG cluster contains only a small number of cells, we observed variation in several TF motifs over pseudotime, including an upregulation of RORB and NFIB motifs, consistent with their respective RNA expression (Figures 3E, 3E′, and 3F). In contrast, the LHX2 motif and RNA expression decrease in the MG cluster (Figures 3E, 3E′, and 3F′).

To better characterize the differences between MPC and MG, we analyzed the genes enriched in the MG cluster (Figures 3G, S2D, and S2E). The scatterplot in Figure 3G shows genes more highly expressed in the MG cluster, such as *NFIA*, *SOX5* and *WTN5B*. In addition, we find an enrichment in Gene Ontology (GO) terms related to "neurogenesis" in the MPC cluster compared with MG (Figure S3C). Taking advantage of the Multiome technique, we next assessed the differences in DNA accessibility between the two clusters. We observed several regions containing higher abundance peaks specific to MG compared with MPC (Figure S3D). For instance, surrounding the *RLBP1* and the *SLC1A3* loci, we identified two accessible regions specific to MG (Figures 3H and 3H′) significantly associated with the expression of these genes. As these two genes (*RLBP1* and *SLC1A3*) are essential for some physiological functions of the MG, this result further confirms the early specification of MG in the human fetal retina. We also investigated two other genes, *SAT1* and *RARB*, which are highly expressed in the MG cluster and that are not known to be human glial-specific genes. We found peaks specific to the MG cluster in regions surrounding these two genes (Figures 3I and 3I′). It thus seems that *SAT1* and *RARB* may be early markers of MG in the human fetal retina. From this analysis, we identified markers that allow us to better discriminate between the MG and the MPC at the DNA, RNA, and protein levels.

Characterization of MG derived from organoids and fetal retina in dissociated cell cultures

We have found that MG are present in the central retina at early stages of fetal human development; however, in the retinal periphery MPC persist as late as FD150 (Figure 1C). Therefore, we isolated MG from stages of retina older than FD150 to reliably establish MG cultures for reprogramming. We tested two different sources of MG: (1) late-stage, pluripotent stem cell-derived retinal organoids (Figure 4A) and (2) fetal retina cultures maintained *in vitro*, which we called "retinospheres." Retinal organoids have been shown to faithfully recapitulate retinogenesis, generating all the retinal cell types with a comparable timeline as the fetal retina (Sridhar et al., 2020). Using MG markers (e.g., RLBP1, SOX2), we find that MG appear between D120 and D136 in retinal organoids (Völkner et al., 2022)

Figure 3. SnRNA-seq and SnATAC-seq show MG specification as early as D59 in the human fetal retina
(A) Feature plots showing the genes RLBP1 and SLC1A3 expression values.
(B) Different phases of the cell cycle, showing that MG are mostly in G1.
(C) Schematic model of progenitor cell fate decisions at FWK8 and FWK10.
(D) Pseudotime values for MG and MPC cells in G1 only.
(E) Heatmap of the enriched TF motifs in DNA accessible regions found in the MG lineage over pseudotime (E′) and the corresponding RNA expression for each TF of the heatmap.
(F) Chromvar scores of NFIB (F) anf LHX2 (F′) motifs.
(G) Scatterplot of the genes expressed differentially between MPC (blue) and MG (red). Genes that show significantly different expression are colored and the top 10 genes are labeled. RLBP1 is significantly more highly expressed in the MG than in the MPCs, but it is not in the top 10 genes.
(H and H′) Peak to gene analysis at the RLBP1 (H) and SLC1A3 (H′) loci. (Left) Feature plots of the peaks highlighted on the right.
(I and I′) Peak to gene analysis for the SAT1 (I) and RARB (I′) loci. (Left) Feature plots of the RNA-seq expression levels. (Middle) Feature plots of the peaks shown on the right, to demonstrate correspondence between the gene expression and accessible chromatin at these additional glial genes.

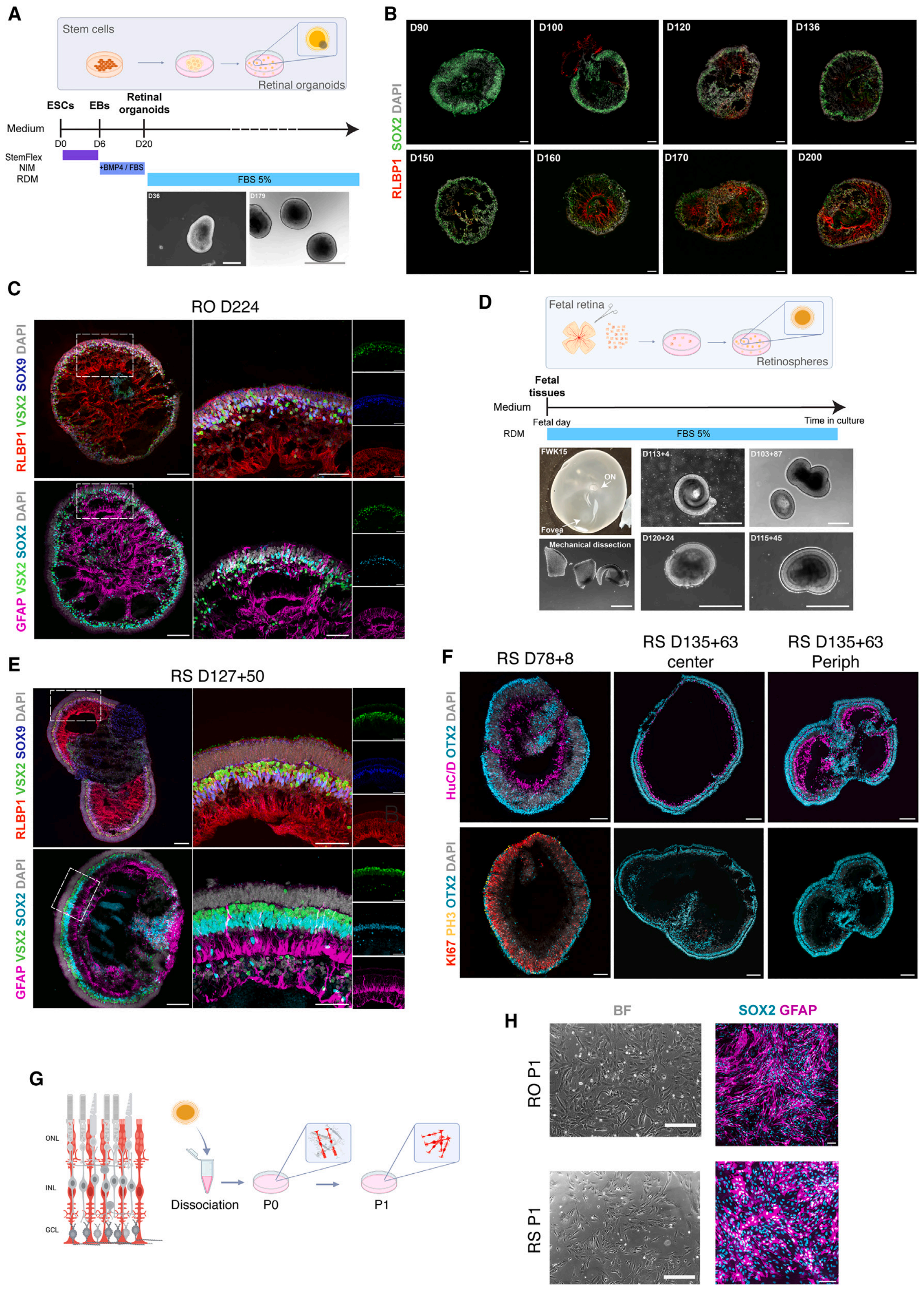

(legend on next page)

(Figure 4B). By 200 days, MG markers (RLBP1, SOX9, SOX2, GFAP, and VSX2) are strongly expressed in all organoids (Figure 4C). Although these cells may not be identical to adult MG, they differ from MPC in their lack of genes associated with neurogenesis such as *ASCL1* and *NEUROG2* (Cowan et al., 2020; Sridhar et al., 2020; Thomas et al., 2022); however, it is important to note that retinal organoids are heterogeneous, and do not mature synchronously (Capowski et al., 2019; Völkner et al., 2016).

Although MG derived from retinal organoids resemble those present in normal retina, fetal retina can provide an alternate source for dissociated MG cultures (Figure 4D). Since we cannot obtain fetal samples from 200 days of gestation, we have developed a long-term *in vitro* culture of human fetal retina, called retinospheres (RS), which allows fetal retinas of all ages to be maintained in 3D organized and laminated structures for many months (Eldred and Reh, 2021; Sridhar et al., 2020). During the time *in vitro*, the retinal cells continue to mature and differentiate, and the MG can be labeled with the same markers used in retinal organoids (Figure 4E). Therefore, we obtained fetal samples from gestational ages of approximately 100 days and maintained these as RS for another 100 days so that they would be equivalent to the organoids. For example, in Figure 4E we show, RS 127 + 50, which is a 127-day-old fetal sample that was dissected and subsequently maintained for 50 days *in vitro* as a RS. At this age, RS are mostly composed of differentiated neurons (OTX2+, HuC/D+) and contain few proliferating MPC, whereas younger spheres contain a higher percentage of Ki67+ and PH3+ cells (Figure 4F). Since RS can be generated from different regions of the retina, we noticed some differences between RS originating from the central (most mature) or peripheral retina (least mature), consistent with the central to peripheral gradient in maturation of the human retina (Figure 4F).

To generate dissociated cell cultures of the MG, we adapted the protocol previously established for dissociated MG from mice (Pollak et al., 2013; Ueki et al., 2012). Retinal organoids or RS were enzymatically dissociated, and the cells plated for up to 10 days prior to passaging (Figure 4G). Most neurons do not survive in these culture conditions, and passaging the cells leads to further loss in surviving neurons. Cells were characterized with IF after passaging (Figure 4H). Although RLBP1, one of the key markers of MG, is downregulated in the dissociated cultures (Couturier et al., 2021; Ning et al., 2022), other markers of glial cells persist (e.g., GFAP and SOX2), confirming the presence of MG in the cultures derived from either retinal organoids or RS (Figure 4H). In dissociated MG cultures from organoids or from RS, the MG proliferate for at least two population doublings in 10 days; the cells can then be stored for later use (Ning et al., 2022). The dissociated cultures of MG from organoids or RS were very similar, exhibiting a large soma and oval nuclei (Figure 4H) (Eastlake et al., 2019).

Although most cells in the cultures derived from the RS were MG, based on their IF for glial proteins, we noticed the presence of PAX2+/SOX2+ cells, which are potentially retinal astrocytes (Stanke et al., 2010). Moreover, immunolabeling of D150 fetal retinal sections shows PAX2+ cells in the ganglion cell layer, providing further evidence of the astrocytic derivation of the PAX2+ cells (Figure S4A). Of note, the astrocyte population is more highly represented in the dissociated cultures from RS, than from organoids (Figures S4B–S4E). Since we can be reasonably confident that the PAX2+/SOX2+ cells in the dissociated cultures are derived from astrocytes, and are not MG, we removed them from further analysis of the snRNA-seq and single cell RNA sequencing (scRNA-seq) datasets.

ASCL1-mediated reprogramming of MG from retinal organoids

Using the organoid and RS-derived dissociated MG cultures, we tested whether *ASCL1* would induce neurogenesis. MG cells derived from retinal organoids were infected with a lentivirus containing ASCL1-IRES-GFP under a cytomegalovirus (CMV) promoter and maintained in culture

Figure 4. MG development in the retinal organoids and retinosheres s and characterization of MG dissociated cultures
(A) Schematic protocol for embryonic stem cell (ESC) differentiation protocol to generate retinal organoids (RO).
(B) Images of MG development in retinal organoids over time labeled with RLBP1 (red), DAPI (grey) and SOX2 (green). Scale bar, 100 μm.
(C) By 200 days, MG are clearly present as shown by glial markers, including RLBP1 (red), VSX2 (green), SOX9 (blue), and SOX2 (cyan). DAPI (gray). Scale bar, 100 μm.
(D) (Top) Schematic protocol for the generation of Retinospheres (RS). (Bottom) RS made from several fetal retinas and cultured for various times as labeled. Scale bar, 500 μm.
(E) Characterization of the MG in RS with the same markers used for (C). Scale bar, 100 μm.
(F) RS maintained *in vitro* for 8 or 63 days to show progressive differentiation of photoreceptors and bipolar cells (OTX2, cyan) and loss in MPC (Ki67, red and PH3, yellow); HuC/D+ (magenta) amacrine cells and RGCs are also labeled. Scale bar, 100 μm.
(G) Schematic protocol for MG dissociation from RS and RO.
(H) Dissociated MG cultures derived from RS and RO and staining with glial markers SOX2 (cyan) and GFAP (magenta) Scale bar, 100 μm. BF, Brightfield. Scale bar, 250 μm.

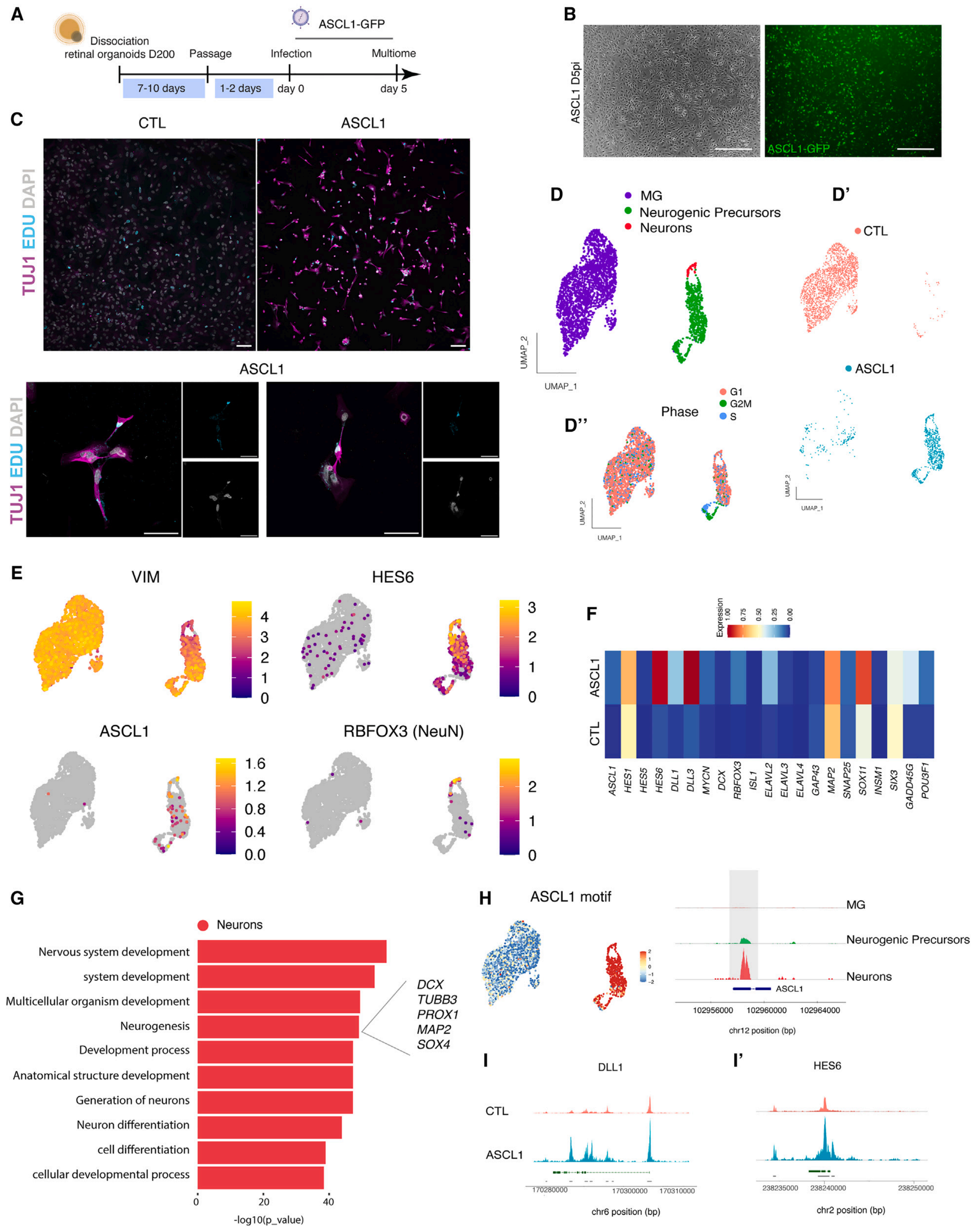

(legend on next page)

for approximately 5 days prior to analysis (Figures 5A and 5B). A lentivirus expressing GFP was used as a control. We assessed the *ASCL1*-expressing MG for evidence of neural reprogramming using IF and Multiome to monitor gene expression and chromatin changes after *ASCL1* over-expression (Figure 5A).

Many cells in the MG cultures infected with *ASCL1* expressed neural markers (Figure 5B), like TUJ1 (*TUBB3*), and acquired a neuronal morphology, while neuronal cells were not observed in the control condition (Figure 5C). The addition of 5-ethynyl-2′-deoxyuridine (EdU) to the culture allowed us to track newly generated cells by incorporation of EdU into newly synthesized DNA. This method allowed us to control for any surviving neurons from the initial dissociation of the organoids. In ASCL1-infected and control conditions, EdU^+ cells were observed; however, we did not observe any $EdU^+/TUJ1^+$ cells in the control condition, whereas we found many examples of $EdU^+/TUJ1^+$ cells in the ASCL1-infected cells (Figure 5C).

The results of ASCL1 infection in MG were further analyzed using snRNA-seq. The UMAP plots of the merged conditions (control [CTL] and ASCL1) show multiple clusters, with cell types identified by their expression of known marker genes (Figure 5D). Both conditions have a large cluster of MG cells (purple), but clusters of neurogenic precursors (green) and neurons (red) were only present in the *ASCL1* treatment condition (Figure 5D′). The cells in the neurogenic precursors cluster express ASCL1 and target genes: *HES6*, *DLL1*, and *DLL3* (Figure 5F). Moreover, a subset of ASCL1-infected cells expresses markers of mitotic proliferation, consistent with prior results in mice that over-expression of *ASCL1* in MG stimulates mitotic proliferation (Figure 5D′′) (Pollak et al., 2013). Importantly, there was a cluster of cells expressing neuronal genes such *RBFOX3* (NeuN), a neural marker (Figure 5E), and a reduction in the expression of glial markers (e.g., vimentin) as the MG transition to neurogenic progenitor and neurons, supporting the assumption that these neurogenic precursor cells are derived from MG (Figure 5E). Compared with the CTL condition, several neuronal genes were upregulated in the ASCL1 condition, including *ELAVL2*, *ELAVL3* (HuC), and *SOX11* (Figure 5F). Furthermore, GO analysis revealed that the terms enriched in the neuronal clusters are associated with “neurogenesis” and “neuron differentiation” (Figure 5G).

These results show that lentiviral expression of *ASCL1* induced neurogenic progenitors and neurons from MG. Since we processed the cells for Multiome analysis, we were able to correlate chromatin accessibility with RNA expression, using Signac. Our data show that the ASCL1 motif is abundant in the clusters induced by the viral expression of ASCL1 (Figure 5H). In addition, *ASCL1* remodels the chromatin to increase accessibility at its predicted targets, such as *HES6* and *DLL1* (Figures 5I and 5I′). These results further demonstrate that human MG can be reprogrammed to neurogenic precursors, with the cells acquiring both a transcriptome and epigenomic states, similar to these cell types observed in the developing retina.

ASCL1-mediated reprogramming of MG cultures from human fetal retina

Although the cells generated in retinal organoids compare very well with those of the fetal retina in transcriptome and developmental timing (Sridhar et al., 2020), long-term cultures of organoids show disorganization of the inner retina that could impact MG development (Capowski et al., 2019). By contrast, the MG in RS maintain their normal structure, even up to 200 days of culture, and therefore may represent a better model for MG *in vitro* (Eldred and Reh, 2021; Sridhar et al., 2020). For this reason, we carried out a similar reprograming experiment to those described above using RS-derived MG. To ensure the MG cultures did not contain any progenitors, RS were treated with a gamma secretase inhibitor (PF4014) for 3 days prior to dissociation of the MG in two-dimensional (2D) cultures. Previous studies have shown that inhibition of the Notch signaling pathway rapidly induces the differentiation of MPC (Chew et al., 2022; Kaufman et al., 2019; Nelson et al., 2007).

To test whether fetal MG can be reprogrammed to neurogenic precursors, we used the same lentiviral-mediated gene delivery for *ASCL1* over-expression and a lentivirus-driving GFP expression as a CTL. Eight days after infection,

Figure 5. ASCL1 induces a neurogenic program in MG derived from organoids *in vitro*
(A) Schematic of the experimental timeline.
(B) ASCL1-infected MG 5 days post infection (D5pi). (Right) Brightfield image. (Left) GFP reporter expression. Scale bar, 500 μm.
(C) Newly generated neurons express TUJ1 (magenta) and EdU+ (cyan) *in vitro* culture after *ASCL1* over-expression. DAPI (gray). Scale bar, 100 μm.
(D) Integrated UMAP plot of the MG cultures (D′) with and without ASCL1. (D′′) UMAP plot showing the different phase of the cell cycle.
(E) Feature plots showing the expression of vimentin (VIM), HES6, ASCL1, and RBFOX3 (NeuN).
(F) Heatmap comparing the average expression of selected genes with and without ASCL1 over-expression (CTL condition).
(G) Top GO biological process analysis for the reprogrammed neuronal cluster.
(H) ASCL1 motif and coverage plot accessibility near *ASCL1*.
(I and I′) Coverage plots of accessibility near (I) *DLL1* and (I′) *HES6* genes.

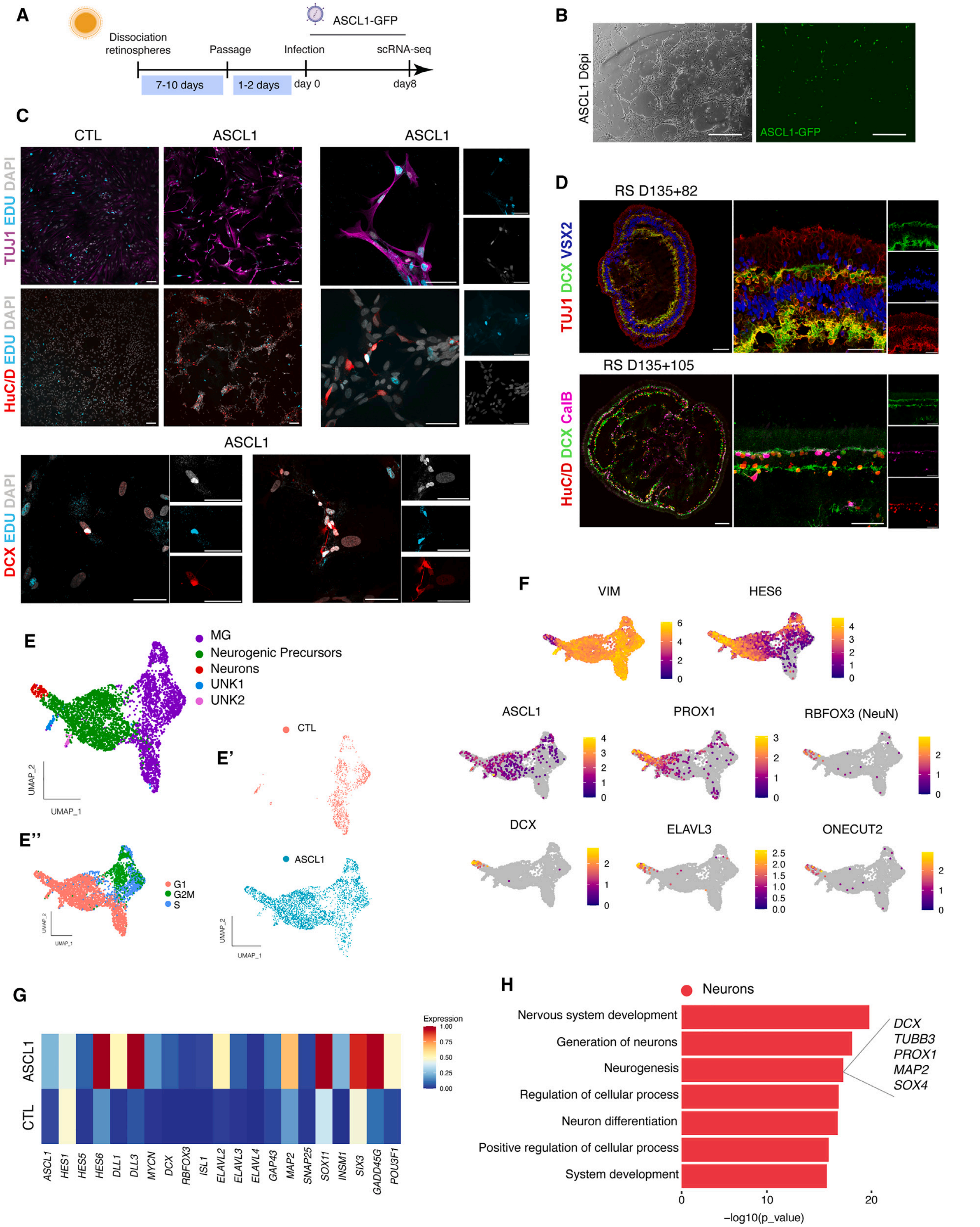

(legend on next page)

we performed IF and scRNA-seq to evaluate the ability of *ASCL1* to reprogram fetal-derived MG (Figures 6A and 6B). The results were similar in the fetal MG to what we had observed in the organoid-derived MG: the ASCL1-infected cells, but not the CTL, contained EdU$^+$ cells that acquired a neuronal morphology and expressed several markers of differentiating neurons, including TUJ1, DCX, and HuC/D (Figure 6C). Although DCX is frequently used as a marker of immature neurons in other regions of the nervous system, this has not been reported for developing retina; therefore, we carried out a parallel analysis of DCX in intact RS to determine the types of neurons that express this gene in the retina. IF analysis demonstrated that DCX overlaps with the neuronal marker TUJ1 (*TUBB3*), the ganglion/amacrine markers HuC/D (*ELAVL3/4*) and calbindin (*CalB*) (Figure 6D). More important, DCX and TUJ1 do not overlap with VSX2, which is expressed in MG, bipolar, and progenitor cells (Figure 6D). Thus, our results demonstrate that expressing *ASCL1* in MG induces a neurogenic program in the cells.

We also analyzed the RS-derived MG with the scRNA-seq approach. Results are shown on UMAP plots and cell types are identified by their expression of known marker genes. In both the CTL and the ASCL1 conditions, we find a cluster of MG (Figure 6E, purple); however, there are several additional clusters that are only present in the ASCL1-infected cells (Figure 6E′). The new clusters are composed of neurogenic precursors (green) and neurons (red), as defined by their expression of genes that identify these cell types (Figure 6E). For example, *HES6* is expressed in neurogenic precursors during retinal development, while *DCX*, *RBFOX3* (NeuN), and *ELAVL3* (HuC) are expressed in newly generated retinal neurons (Figures 6E and 6F). After the addition of the pro-neural TF, we found the activation of key genes downstream of *ASCL1* such as *DLL3*, *INSM1*, and *SOX11* (Figure 6G). This result was then further confirmed with a GO analysis, showing that genes in the neuronal cluster are associated with terms including “neurogenesis” and “neuron differentiation” (Figure 6H). In addition, we also used the top 25 genes expressed in the induced neuronal clusters (Figure 6E) and plotted those onto human fetal retinal Multiome data (Figure 2B): the majority of the top 25 genes are also expressed by cells present in the Npre and the immature RGC cluster in the human fetal retina (Figures S5A and S5B).

Overall, our data show that *ASCL1* can reprogram fetal MG in dissociated cultures, much like the organoid-derived MG (Figures S5C–S5F). To directly compare the reprogramming of MG derived from the two sources, we integrated the data to a single UMAP plot (Figure S6G). This analysis revealed that both sources of human MG (retinal organoids and fetal retinas) can be reprogrammed to neurogenic precursors after *ASCL1* over-expression with similar results (Figures S5G′ and S5G″). However, in both conditions the majority of the reprogrammed cells remained in a neurogenic precursor state, and only a subset differentiate into neurons. One possibility is that Notch signaling, induced by *ASCL1* expression, prevents differentiation into neurons. Examining the chromatin landscape of the reprogrammed cells, we find that, while *ASCL1* activates its downstream targets, feedback inhibitors of *ASCL1*, such as *ID1*, *ID3*, and *HES1*, are also expressed in these cells (Figure S6A) (Boareto et al., 2017; Sueda et al., 2019; Sueda and Kageyama, 2020; Viñals et al., 2004). These results suggest that inhibition of Notch signaling may increase the number of new neurons generated by the ASCL1 reprogrammed MG. To test this hypothesis, we used our *in vitro* regenerative paradigm with the addition of a Notch inhibitor in the dissociated MG cultures (PF4014 a gamma secretase inhibitor) 4 days after the *ASCL1* induction and performed scRNA-seq at day 6 (Figure S6B). Consistent with our previous data, cells follow the same reprogramming trajectory and expressed the same neuronal markers (*DCX*, *ELAVL3*, and *RBFOX3*) as previously demonstrated (Figures S6C and S6D). Although the neurogenic efficiency is slightly increased after the addition of the Notch inhibitor treatment compared with the ASCL1 condition alone, the difference is relatively small (Figures S6E and S6F). However, we observed a cluster of OTX2+-induced neurons only in the Notch inhibition condition (Figure S6G). Overall, this analysis revealed that Notch inhibition may impact cell fate after *ASCL1* expression in human MG cultures.

Figure 6. ASCL1 induces a neurogenic program in MG derived from human fetal retina *in vitro*
(A) Schematic of the experimental timeline.
(B) ASCL1-infected MG 6 days post infection (D6pi). (Right) Brightfield image. (Left) GFP reporter expression. Scale bar, 500 μm.
(C) Newly generated neurons express TUJ1 (magenta), HuC/D (red, top), DCX (red, bottom), and EdU+ (cyan) *in vitro* culture after ASCL1 over-expression. DAPI is in gray. Scale bar, 100 μm. MG-reprogrammed neurons with higher magnification. Scale bar, 50 μm.
(D) RS sections showing that DCX (green) co-localized with TUJ1 (red, top), HuC/D (red, bottom), and calbindin (magenta, bottom), but not with VSX2 (blue, top). Scale bar, 100 μm for composite images. Scale bar, 50 μm for the individual channels.
(E) Integrated UMAP plot of the MG cultures (E′) with and without ASCL1. (E″) UMAP plot showing the different phase of the cell cycle.
(F) Feature plots showing the expression of vimentin (VIM), HES6, ASCL1, PROX1, DCX, RBFOX3 (NeuN), ELAVL3, and ONECUT2.
(G) Heatmap comparing the average expression of selected genes in the two conditions (CTL and ASCL1 over-expression).
(H) Top GO biological process analysis for the reprogrammed neuronal cluster.

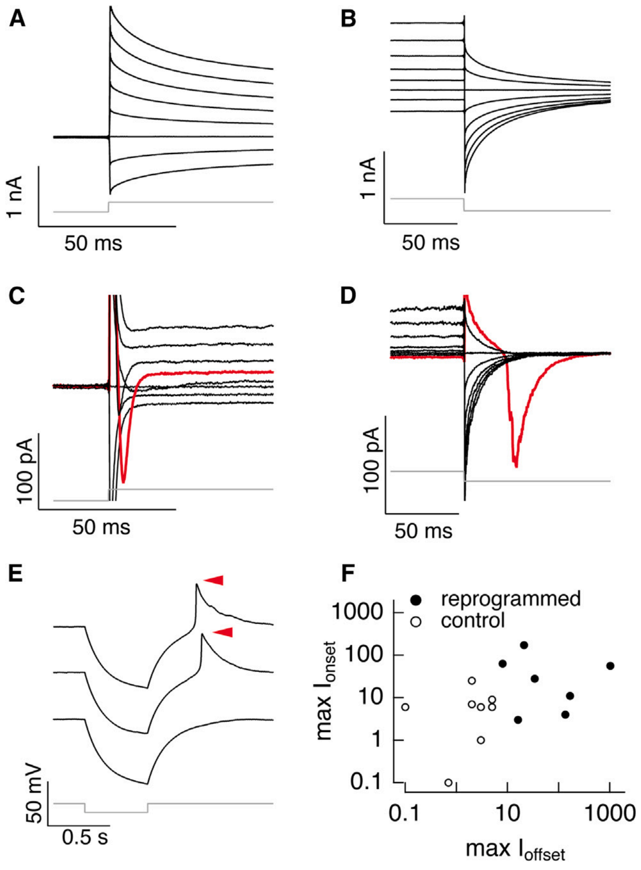

Figure 7. MG-derived cells demonstrate electrophysiological properties *in vitro*

Voltage-activated conductances in reprogrammed cells.

(A and B) Current responses produced by voltage steps ranging from −100 mV to +40 mV from a starting voltage of −60 mV. (A) Responses at step onset and (B) responses at step offset.

(C and D) Current responses for two different reprogrammed cells to the same protocol as in (A) and (B). The red trace in (C) was produced for a step from −60 mV to −20 mV, and the red trace in (D) for a step from −60 mV to −100 mV.

(E) Voltage responses elicited by a hyperpolarizing current step. The three traces shown are individual responses to the same current step. This cell generated all-or-none depolarizations for these steps (red arrows).

(F) Summary of inward currents produced in CTL and reprogrammed cells at the onset or offset of voltage steps. Each point represents the maximal inward currents at step onset plotted against that at step offset for a single cell.

MG-derived cells demonstrate neuronal electrophysiological properties

We then used electrophysiology to characterize the electrical properties of reprogrammed cells derived from retinosphere MG. Patch-clamp electrophysiology was performed on GFP+ cells 6 days after *ASCL1* over-expression. We recorded changes in membrane voltage in response to injected current steps and changes in current in response to voltage steps. The electrical properties of CTL and reprogrammed cells differed substantially. Figures 7A and 7B present current responses of a CTL cell, with Figure 7A showing currents shortly after a series of depolarizing voltage steps and Figure 7B showing responses at the end of the voltage steps. The near uniform spacing of the current traces suggests at most a modest contribution of voltage-dependent conductance. This behavior is more consistent with a glial phenotype than a neuronal phenotype. In some cells, however, depolarization opened ion channels, as demonstrated by an increased spacing of the current responses; these currents were likely generated by voltage-activated K^+ channels. Figures 7C and 7D demonstrate responses of reprogrammed cells to the same protocol; responses at step onset (Figure 7C) and offset (Figure 7D) are from different cells. Six of seven reprogrammed cells displayed clear inward currents at the onset or offset of voltage steps (red traces in Figures 7C and 7D). These inward currents produced by the reprogrammed cells likely originate from Na^+ or Ca^{2+} currents, consistent with a neuronal phenotype. Indeed, injecting currents into some of the reprogrammed cells produced amplified depolarizing voltage changes—and in some cases full all-or-none action potentials (Figure 7E).

There was considerable heterogeneity in the magnitude of the inward currents across reprogrammed cells. Hence to summarize these results, we plotted the maximum inward currents for each individual CTL or reprogrammed cell at the onset (y axis) and offset (x axis) of the voltage steps (Figure 7F, each point represents one cell). Reprogrammed cells generated larger inward currents, often by a factor of 10–100. Overall, our results show that *ASCL1*-reprogrammed cells display electrophysiological properties quite unlike glia and more similar to immature neurons, consistent with the scRNA-seq and IF data.

DISCUSSION

Recent studies from our group and others have shown that MG in mouse retina can be stimulated to regenerate new neurons after injury by over-expressing *Ascl1*, a pro-neural TF, along with the histone deacetylase (HDAC) inhibitor: trichostatin A (TSA); however, the feasibility of this strategy for human cells was not known when we began our studies (Jorstad et al., 2017; 2020; Todd et al., 2021; 2022). The findings of the current report show that human MG, derived from either organoids or fetal retina, can be reprogrammed to a neurogenic state using *ASCL1*, much like MG from mice.

In our detailed study of MG development in the human fetal retina, we have found that the first MG appear earlier

than previously thought; they are already present in the PF at FD59 (Hoshino et al., 2017; Lu et al., 2020; Sridhar et al., 2020). MG soon appear outside the fovea and spread across the retina, but do not yet reach the periphery until after FD150, the oldest fetal age we have been able to examine. Nevertheless, a recently developed culture system, RS, allows us to maintain fetal retina for hundreds of days, allowing for MG maturation (Eldred and Reh, 2021; Sridhar et al., 2020). Using this system, we find that, by 180 days, RLBP1-expressing MG are present in all retinospheres. Retinal organoids follow a similar time course, and, by 200 days, organoids contain MG that express mature glial markers such as RLBP1 (Ning et al., 2022; Sridhar et al., 2020; Völkner et al., 2022).

To develop the conditions for culturing MG as dissociated cells, we relied on previously published methods in mice (Pollak et al., 2013). Like mouse MG cultures, human MG display a glial morphology with prominent oval nuclei (Ning et al., 2022). Dissociated MG cultures have several potential advantages over the explant cultures or organoids, in that they can be expanded and banked for later use. In addition, dissociated cell cultures are easier to infect with viral reprogramming factors. However, some key genes normally present in MG, such as *RLBP1*, are downregulated in dissociated cell cultures (Couturier et al., 2021; Ning et al., 2022). Changes in the cellular environment combined with a loss of polarity of the MG in dissociated culture may explain this difference, although these changes were not observed in mouse MG cultures. Nevertheless, dissociated human MG are still easily identifiable in dissociated culture, as they express others glial marker such as SOX2, PAX6, and GFAP that are not present in other potentially contaminating cell types, such as astrocytes.

We find that *ASCL1* over-expression induces neurogenesis in the human MG cultures. Using IF, we showed that induced neurons exhibit a neuronal morphology: a small, round nucleus and multiple, long processes. Furthermore, they express the pan-neuronal marker TUJ1 (*TUBB3*), in addition to DCX and HuC/D, which are both expressed in RGC and amacrine cells in the human fetal retina. scRNA-seq analysis combined with electrophysiological studies further validated this result, showing that the MG-derived neurons express the same neural markers previously identified by IF (DCX, HuC/D [*ELAVL3/4*]) and display neuronal characteristics such as Na^{+} and Ca^{2+} currents and action potentials. Overall, our data suggest that *ASCL1* induces human fetal MG neurogenesis toward immature RGC-like and amacrine-like neurons *in vitro*.

The types of neurons generated by MG in human cultures differs from what we observe in mice, where *Ascl1* induced MG to generate Otx2+ bipolar-like cells (Jorstad et al., 2017; Pollak et al., 2013; Ueki et al., 2015). It remains unclear why there are differences in the types of neurons generated by the reprogrammed MG from mouse and human. It is possible that *ASCL1* plays a different role in neuronal specification in the two species; however, single-cell transcriptomics and epigenomics show similar patterns of expression and motif accessibility for *ASCL1* in human and mouse development (Hoshino et al., 2017; Jorstad et al., 2020; VandenBosch et al., 2020). Alternatively, it is possible that differences in the duration of the cultures affects the types of neuron fate choices available with *ASCL1* over-expression. For example, the mouse MG are cultured for only 1–2 weeks, while the human cells were *in vitro* for 200 days. Although both organoid-derived and RS-derived MG are quite similar to freshly isolated fetal MG, dissociation and passaging may cause them to diverge in gene expression over time; some of the genes downregulated in MG could play a role in *OTX2* induction after *ASCL1* over-expression. It is also intriguing that inhibition of Notch signaling enables the generation of *OTX2*+ neurons. The bias toward the OTX2 lineage after Notch signaling inhibition has already been described in previous studies (Chew et al., 2022; Finkbeiner et al., 2022; Jadhav et al., 2006).

Although the addition of a Notch inhibitor slightly increases the neuronal cluster compared with the ASCL1 only condition, the majority of the cells remain in the neurogenic precursor fate. Additionally, other factors have been shown to limit neurogenesis from ASCL1-reprogrammed MG in mice, such as microglial ablation or STAT inhibition (Jorstad et al., 2020; Todd et al., 2020). Testing these variables in human MG reprograming paradigm may lead to increased neurogenesis. Furthermore, additional TFs may be needed to increase the efficiency of neurogenesis *in vitro*, including the bHLH TF ATOH7 (Todd et al., 2021; 2022). Previous reports in mice have also found that reprogramming MG with *Ascl1 in vivo* results in a more stable and well differentiated population of MG-derived neurons than what occurs with *in vitro* reprogramming with the same factor (Pollak et al., 2013; Ueki et al., 2015). Therefore, further studies in a more intact environment, such as the 3D RS and retinal organoids, will be needed to further test the reprogramming potential of *ASCL1* in human MG.

Taken together, our work shows evidence of regenerative capacity in human MG. This study constitutes a proof of principle that the human MG, as well as the mouse MG, can be reprogrammed into neurons after the over-expression of the pro-neural TF *ASCL1*. In the context of restoring vision loss, cell transplantation and regenerative strategies have the potential to restore lost neurons and represent complementary approaches. However, while stimulation

of regeneration may someday be a viable strategy to repair the human retina, many challenges remain. We anticipate that future studies will continue to build on our findings, to explore the regenerative capacities of adult MG in degenerative contexts.

EXPERIMENTAL PROCEDURES

Resource availability

Data are available in the main text or in the supplemental information. All unique reagents generated for this study are available from the lead contact without restriction.

Corresponding author

Further information and requests for resources and reagents should be directed to and will be fulfilled by the corresponding author Thomas A. Reh: tomreh@uw.edu.

Materials availability

No new reagents were generated for this report.

Data and code availability

All the Multiome and scRNA-seq datasets generated for this manuscript have been deposited on the Gene Expression Omnibus (GEO) repository under the accession number GSE246169.

Organoid cultures

Retinal organoids were generated as previously demonstrated (Meyer et al., 2009; Sridhar et al., 2020; Zhong et al., 2014). Detailed protocol is available in the supplemental information.

Fetal retina tissue and RS cultures

Human retinal tissues were obtained from the Birth Defect Research Laboratory at the University of Washington using an approved protocol (UW5R24HD000836). Sample ages were estimated by different techniques, including gestational ultrasound examination, crown-rump length, and fetal foot length (Fitzsimmons et al., 1994). RS were made as previously described (Sridhar et al., 2020).

Plasmids and viral production for 2D MG cultures infection

PLOC-hASCL1-IRES-turboGFP(nuc) (Open Biosystems) and PLOC-IRES-turboGFP(nuc) plasmids were used as previously described (Pollak et al., 2013). Lentiviral particles containing the plasmid constructions were produced using Lenti-Xtm Packing Single Shots (VSV-G), according to the manufacturer's protocol (Takara Bio).

Cell dissociation and MG cultures

Retinal organoids and RS were digested with Papain (Worthington) for 15–30 min at 37°C, on a nutator with mechanical dissociation using a P1000 pipette every 10 min. Ovomucin (Worthington) was added to the solution to stop the reaction. The suspension was then spun down for 7 min at 300 RPM, 4°C. Supernatant was then removed, and cells were resuspended with the appropriate volume of medium (RDM 5%) depending on the experiments. Culture medium was changed every other day until the cells reached confluency. MG cultures were next passaged and treated with lentivirus to overexpress *ASCL1*.

IF

Retinal organoids and RS were fixed with cold PFA 4% for 15 min and then washed three times with PBS. Fixed tissues were embedded in 10%, 20%, or 30% sucrose overnight at 4°C. Tissues were frozen in OCT and cryosectioned at 15 μm. Cultured MG were plated on glass coverslips treated with poly-D-lysine and coated with Matrigel. Cells were fixed with cold PFA 4% for 15 min and then washed three times with PBS. A blocking solution containing 10% horse serum, 90% PBS, and 0.5% Triton X-100 was then used for 1 h at room temperature (RT). Primary antibodies were diluted in the blocking solution (Table S1) overnight at 4°C. The next day, cells were washed three times with PBS before adding the secondary blocking solution containing the blocking solution, 1/8,000 DAPI, and the secondary antibodies (Table S1) for 1 h, RT. Cells were then washed three times with PBS, and sections or coverslips were mounted using fluoromount-G (SouthernBiotech) medium. For EdU labeling, cells were incubated for 30 min at RT with the Click-it solution (Click-iT EdU Assay, Invitrogen) and then washed three times with PBS before being mounted.

Whole mount staining and clearing protocol

The clearing protocol was carried out using the EyeDisco protocol as previously described (Vigouroux et al., 2020).

Three-dimensional LSFM imaging

Cleared, agarose-embedded, intact fetal eye samples were imaged with a light sheet microscope (SmartSPIM, LifeCanvas Technologies) using a 3.6× objective (ThorLabs, TL4X-SAP, NA = 0.2) with lateral sampling of 1.8 μm/pixel in XY and 2-μm steps in Z. The samples were submerged and imaged in dibenzyl ether (Sigma Aldrich) with a refractive index of 1.562. A single laser sheet was used at 30% power for 488-nm, 50% power for 561-nm, and 10% power for 639-nm wavelengths. Each channel had an acquisition exposure time of 2 ms. The resulting raw image arrays (2 × 2) were stitched using commercial stitching software (LifeCanvas Technologies). Then, 2D and 3D images were rendered using Arivis Vision4D v3.6.2 (Zeiss) software.

Electrophysiology

Recordings were performed on dissociated cells in the treated and CTL condition. Treated cells were infected with a HES1-ASCL1-GFP lentivirus and CTL cells were infected with the HES1-GFP lentivirus (Sueda et al., 2019). Viruses were generated by Vector Builder. Whole-cell patch clamp recordings were made with a K-based internal solution containing 123 mM K-aspartate, 10 mM KCl, 10 mM HEPES, 1 mM $MgCl_2$, 1 mM $CaCl_2$, 2 mM EGTA, 4 mM Mg-ATP, 0.5 mM Tris-GTP, and 0.1 mM Alexa (555). Cells expressing GFP were targeted for recording, and cell identity was confirmed by imaging the Alexa 555 after recording. Whole-cell patch pipettes had resistances of 12–14 MΩ. Access resistance was <25 MΩ for all cells. Reported voltages have not been corrected for an approximately −10 mV liquid junction potential.

Single-cell RNA construction

Cells were harvested as previously described (Todd et al., 2022). Briefly, cells were passed through a 35-μm strainer and loaded to 10× genomics chip type G. Library constructions were performed using the Chromium Next GEM single Cell 3′ Reagent kits v3.1 (Dual Index) according to the manufacturers' instructions. Cells were next encapsulated in a gel and received a unique barcode using the 10× chromonium controller. Further information is available in the Supplemental information.

SUPPLEMENTAL INFORMATION

Supplemental information can be found online at https://doi.org/10.1016/j.stemcr.2023.10.021.

ACKNOWLEDGMENTS

We thank Dr. Ian Glass at the BDRL (Birth Defect Research Laboratory) for their help with the human fetal tissues. Fetal retina tissue samples with no identifiers were made available through the Birth Defects Research Laboratory at University of Washington (UW5R24HD000836). We also would like to thank all the members of the Reh lab and the Bermingham-McDonogh lab for their valuable comments on the manuscript. We thank Dr. Don Zack, Johns Hopkins University, for his gift of the H7 BRN3-tdTomato cell line. This work is funded by a grant from the Foundation Fighting Blindness (TA-RM-0620-0788-UWA) to T.A.R. and by a sponsored research agreement with Tenpoint Therapeutics.

AUTHOR CONTRIBUTIONS

Overall conceptualization, J.W. and T.A.R.; organoid and retinosphere cell culture, J.W., D.H., F.K., and A.K.H.; tissue acquisition and processing, J.W., D.H., F.K., and A.K.H.; immunohistochemistry and microscopy, J.W., D.H., F.K., A.K.H., S.A.G., and A.D.M.; 10× single cell genomics data analysis: J.W., C.F., A.P, I.O.L., and T.A.R.; electrophysiology experiments, F.R.; manuscript preparation and writing, J.W. and T.A.R.; funding acquisition, T.A.R.; and supervision and project administration, T.A.R.

DECLARATION OF INTERESTS

This research is funded in part by a sponsored research agreement with Tenpoint Therapeutics; T.A.R. is a co-founder and consultant. Some of the findings in this manuscript are part of a patent that has been submitted by the University of Washington: PCT/US23/65219.

Received: March 22, 2023
Revised: October 27, 2023
Accepted: October 30, 2023
Published: November 30, 2023

REFERENCES

Boareto, M., Iber, D., and Taylor, V. (2017). Differential interactions between Notch and ID factors control neurogenesis by modulating Hes factor autoregulation. Development (Camb.) *144*, 3465–3474. https://doi.org/10.1242/dev.152520.

Bringmann, A., Iandiev, I., Pannicke, T., Wurm, A., Hollborn, M., Wiedemann, P., Osborne, N.N., and Reichenbach, A. (2009). Cellular signaling and factors involved in Müller cell gliosis: Neuroprotective and detrimental effects. Prog. Retin. Eye Res. *28*, 423–451. https://doi.org/10.1016/j.preteyeres.2009.07.001.

Capowski, E.E., Samimi, K., Mayerl, S.J., Phillips, M.J., Pinilla, I., Howden, S.E., Saha, J., Jansen, A.D., Edwards, K.L., Jager, L.D., et al. (2019). Reproducibility and staging of 3D human retinal organoids across multiple pluripotent stem cell lines. Development (Camb.) *146*, dev171686. https://doi.org/10.1242/dev.171686.

Cepko, C.L., Austin, C.P., Yang, X., Alexiades, M., and Ezzeddine, D. (1996). Cell Fate Determination in the Vertebrate Retina, *93*.

Chew, S.H., Martinez, C., Chirco, K.R., Kandoi, S., and Lamba, D.A. (2022). Timed Notch Inhibition Drives Photoreceptor Fate Specification in Human Retinal Organoids. Invest. Ophthalmol. Vis. Sci. *63*, 12. https://doi.org/10.1167/iovs.63.10.12.

Couturier, A., Blot, G., Vignaud, L., Nanteau, C., Slembrouck-Brec, A., Fradot, V., Acar, N., Sahel, J.A., Tadayoni, R., Thuret, G., et al. (2021). Reproducing diabetic retinopathy features using newly developed human induced-pluripotent stem cell-derived retinal Müller glial cells. Glia *69*, 1679–1693. https://doi.org/10.1002/glia.23983.

Cowan, C.S., Renner, M., de Gennaro, M., Gross-Scherf, B., Goldblum, D., Hou, Y., Munz, M., Rodrigues, T.M., Krol, J., Szikra, T., et al. (2020). Cell Types of the Human Retina and Its Organoids at Single-Cell Resolution. Cell *182*, 1623–1640.e34. https://doi.org/10.1016/j.cell.2020.08.013.

Dyer, M., and Cepko, C. (2000). Control of Müller glial cell proliferation and activation following retinal injury. Nature *3*, 873–880.

Eastlake, K., Wang, W., Jayaram, H., Murray-Dunning, C., Carr, A.J.F., Ramsden, C.M., Vugler, A., Gore, K., Clemo, N., Stewart, M., et al. (2019). Phenotypic and Functional Characterization of Müller Glia Isolated from Induced Pluripotent Stem Cell-Derived Retinal Organoids: Improvement of Retinal Ganglion Cell Function upon Transplantation. Stem Cells Transl. Med. *8*, 775–784. https://doi.org/10.1002/sctm.18-0263.

Eldred, K.C., Hadyniak, S.E., Hussey, K.A., Brenerman, B., Zhang, P.W., Chamling, X., Sluch, V.M., Welsbie, D.S., Hattar, S., Taylor, J., et al. (2018). Thyroid hormone signaling specifies cone subtypes in human retinal organoids. Science *362*, eaau6348. https://doi.org/10.1126/science.aau6348.

Eldred, K.C., and Reh, T.A. (2021). Human retinal model systems: Strengths, weaknesses, and future directions. Dev. Biol. *480*, 114–122. https://doi.org/10.1016/j.ydbio.2021.09.001.

Fausett, B.v., Gumerson, J.D., and Goldman, D. (2008). The proneural basic helix-loop-helix gene Ascl1a is required for retina regeneration. J. Neurosci. *28*, 1109–1117. https://doi.org/10.1523/JNEUROSCI.4853-07.2008.

Finkbeiner, C., Ortuño-Lizarán, I., Sridhar, A., Hooper, M., Petter, S., and Reh, T.A. (2022). Single-cell ATAC-seq of fetal human retina and stem-cell-derived retinal organoids shows changing chromatin landscapes during cell fate acquisition. Cell Rep. *38*, 110294. https://doi.org/10.1016/j.celrep.2021.110294.

Fisher, A.J., and Reh, T.A. (2001). Müller Glia Are a Potential Source of Neural Regeneration in the Postnatal Chicken Retina. https://doi.org/10.1038/85090.

Fitzsimmons, J., Fantelc, A., and Shepard, T.H. (1994). Growth parameters in mid-trimester fetal Turner syndrome. Early Human Development, *38*.

Goldman, D. (2014). Müller glial cell reprogramming and retina regeneration. Nat. Rev. Neurosci. *15*, 431–442. https://doi.org/10.1038/nrn3723.

Hendrickson, A. (2016). Development of Retinal Layers in Prenatal Human Retina. Am. J. Ophthalmol. *161*, 29–35.e1. https://doi.org/10.1016/j.ajo.2015.09.023.

Hendrickson, A.E., and Yuodelis, C. (1984). The Morphological Development of the Human Fovea. Ophthalmology *91*, 603–612. https://doi.org/10.1016/S0161-6420(84)34247-6.

Hendrickson, A., Possin, D., Vajzovic, L., and Toth, C.A. (2012). Histologic Development of the Human Fovea From Midgestation to Maturity. Am. J. Ophthalmol. *154*, 767–778.e2. https://doi.org/10.1016/j.ajo.2012.05.007.

Hoang, T., Wang, J., Boyd, P., Wang, F., Santiago, C., Jiang, L., Yoo, S., Lahne, M., Todd, L.J., Jia, M., et al. (2020). Gene regulatory networks controlling vertebrate retinal regeneration. Science *370*, eabb8598. https://doi.org/10.1126/science.abb8598.

Hoshino, A., Ratnapriya, R., Brooks, M.J., Chaitankar, V., Wilken, M.S., Zhang, C., Starostik, M.R., Gieser, L., La Torre, A., Nishio, M., et al. (2017). Molecular Anatomy of the Developing Human Retina. Dev. Cell *43*, 763–779.e4. https://doi.org/10.1016/j.devcel.2017.10.029.

Hu, Y., Wang, X., Hu, B., Mao, Y., Chen, Y., Yan, L., Yong, J., Dong, J., Wei, Y., Wang, W., et al. (2019). Dissecting the transcriptome landscape of the human fetal neural retina and retinal pigment epithelium by single-cell RNA-seq analysis. PLoS Biol. *17*, e3000365. https://doi.org/10.1371/journal.pbio.3000365.

Jadhav, A.P., Mason, H.A., and Cepko, C.L. (2006). Notch 1 inhibits photoreceptor production in the developing mammalian retina. Development *133*, 913–923. https://doi.org/10.1242/dev.02245.

Jorstad, N.L., Wilken, M.S., Grimes, W.N., Wohl, S.G., Vandenbosch, L.S., Yoshimatsu, T., Wong, R.O., Rieke, F., and Reh, T.A. (2017). Stimulation of functional neuronal regeneration from Müller glia in adult mice. Nature *548*, 103–107. https://doi.org/10.1038/nature23283.

Jorstad, N.L., Wilken, M.S., Todd, L., Finkbeiner, C., Nakamura, P., Radulovich, N., Hooper, M.J., Chitsazan, A., Wilkerson, B.A., Rieke, F., and Reh, T.A. (2020). STAT Signaling Modifies Ascl1 Chromatin Binding and Limits Neural Regeneration from Muller Glia in Adult Mouse Retina. Cell Rep. *30*, 2195–2208.e5. https://doi.org/10.1016/j.celrep.2020.01.075.

Kaufman, M.L., Park, K.U., Goodson, N.B., Chew, S., Bersie, S., Jones, K.L., Lamba, D.A., and Brzezinski, J.A. (2019). Transcriptional profiling of murine retinas undergoing semi-synchronous cone photoreceptor differentiation. Dev. Biol. *453*, 155–167. https://doi.org/10.1016/j.ydbio.2019.05.016.

Lu, Y., Shiau, F., Yi, W., Lu, S., Wu, Q., Pearson, J.D., Kallman, A., Zhong, S., Hoang, T., Zuo, Z., et al. (2020). Single-Cell Analysis of Human Retina Identifies Evolutionarily Conserved and Species-Specific Mechanisms Controlling Development. Dev. Cell *53*, 473–491.e9. https://doi.org/10.1016/j.devcel.2020.04.009.

Lyu, P., Hoang, T., Santiago, C.P., Thomas, E.D., Timms, A.E., Appel, H., Gimmen, M., Le, N., Jiang, L., Kim, D.W., et al. (2021). Gene regulatory networks controlling temporal patterning, neurogenesis, and cell-fate specification in mammalian retina. Cell Rep. *37*, 109994. https://doi.org/10.1016/j.celrep.2021.109994.

Meyer, J.S., Shearer, R.L., Capowski, E.E., Wright, L.S., Wallace, K.A., McMillan, E.L., Zhang, S.C., and Gamm, D.M. (2009). Modeling early retinal development with human embryonic and induced pluripotent stem cells. Proc. Natl. Acad. Sci. USA *106*, 16698–16703. https://doi.org/10.1073/pnas.0905245106.

Nelson, B.R., Hartman, B.H., Georgi, S.A., Lan, M.S., and Reh, T.A. (2007). Transient inactivation of Notch signaling synchronizes differentiation of neural progenitor cells. Dev. Biol. *304*, 479–498. https://doi.org/10.1016/j.ydbio.2007.01.001.

Ning, R., Zheng, D., Xie, B., Gao, G., Xu, J., Xu, P., Wang, Y., Peng, F., Jiang, B., Ge, J., and Zhong, X. (2022). Spatial and Temporal Development of Müller Glial Cells in hiPSC-Derived Retinal Organoids Facilitates the Cell Enrichment and Transcriptome Analysis. Front. Cell. Neurosci. *16*, 820396. https://doi.org/10.3389/fncel.2022.820396.

Pollak, J., Wilken, M.S., Ueki, Y., Cox, K.E., Sullivan, J.M., Taylor, R.J., Levine, E.M., and Reh, T.A. (2013). ASCL1 reprograms mouse Müller glia into neurogenic retinal progenitors. Development (Camb.) *140*, 2619–2631. https://doi.org/10.1242/dev.091355.

Reichenbach, A., and Bringmann, A. (2020). Glia of the human retina. Glia *68*, 768–796. https://doi.org/10.1002/glia.23727.

Salman, A., McClements, M.E., and Maclaren, R.E. (2021). Insights on the regeneration potential of müller glia in the mammalian retina. Cells *10*, 1957. https://doi.org/10.3390/cells10081957.

Sridhar, A., Hoshino, A., Finkbeiner, C.R., Chitsazan, A., Dai, L., Haugan, A.K., Eschenbacher, K.M., Jackson, D.L., Trapnell, C., Bermingham-McDonogh, O., et al. (2020). Single-Cell Transcriptomic Comparison of Human Fetal Retina, hPSC-Derived Retinal Organoids, and Long-Term Retinal Cultures. Cell Rep. *30*, 1644–1659.e4. https://doi.org/10.1016/j.celrep.2020.01.007.

Stanke, J., Moose, H.E., El-Hodiri, H.M., and Fischer, A.J. (2010). Comparative study of Pax2 expression in glial cells in the retina and optic nerve of birds and mammals. J. Comp. Neurol. *518*, 2316–2333. https://doi.org/10.1002/cne.22335.

Stein, J.D., Khawaja, A.P., and Weizer, J.S. (2021). Glaucoma in Adults - Screening, Diagnosis, and Management: A Review. JAMA, J. Am. Med. Assoc. *325*, 164–174. https://doi.org/10.1001/jama.2020.21899.

Sueda, R., Imayoshi, I., Harima, Y., and Kageyama, R. (2019). High Hes1 expression and resultant Ascl1 suppression regulate quiescent vs. active neural stem cells in the adult mouse brain. Genes Dev. *33*, 511–523. https://doi.org/10.1101/gad.323196.118.

Sueda, R., and Kageyama, R. (2020). Regulation of active and quiescent somatic stem cells by Notch signaling. Dev. Growth Differ. *62*, 59–66. https://doi.org/10.1111/dgd.12626.

Thomas, E.D., Timms, A.E., Giles, S., Harkins-Perry, S., Lyu, P., Hoang, T., Qian, J., Jackson, V.E., Bahlo, M., Blackshaw, S., et al. (2022). Cell-specific cis-regulatory elements and mechanisms of

non-coding genetic disease in human retina and retinal organoids. Dev. Cell *57*, 820–836.e6. https://doi.org/10.1016/j.devcel.2022.02.018.

Todd, L., Finkbeiner, C., Wong, C.K., Hooper, M.J., and Reh, T.A. (2020). Microglia Suppress Ascl1-Induced Retinal Regeneration in Mice. Cell Rep. *33*, 108507. https://doi.org/10.1016/j.celrep.2020.108507.

Todd, L., Hooper, M.J., Haugan, A.K., Finkbeiner, C., Jorstad, N., Radulovich, N., Wong, C.K., Donaldson, P.C., Jenkins, W., Chen, Q., et al. (2021). Efficient stimulation of retinal regeneration from Müller glia in adult mice using combinations of proneural bHLH transcription factors. Cell Rep. *37*, 109857. https://doi.org/10.1016/j.celrep.2021.109857.

Todd, L., Jenkins, W., Finkbeiner, C., Hooper, M.J., Donaldson, P.C., Pavlou, M., Wohlschlegel, J., Ingram, N., Rieke, F., Reh, T.A., and Mu, X. (2022). Reprogramming Müller glia to regenerate ganglion-like cells in adult mouse retina with developmental transcription factors. Sci. Adv. *8*, eabq7219. https://doi.org/10.1126/sciadv.abq7219.

Todd, L., and Reh, T.A. (2022). Comparative Biology of Vertebrate Retinal Regeneration: Restoration of Vision through Cellular Reprogramming. Cold Spring Harbor Perspect. Biol. *14*, a040816. https://doi.org/10.1101/CSHPERSPECT.A040816.

Tworig, J.M., and Feller, M.B. (2022). Müller Glia in Retinal Development: From Specification to Circuit Integration. Front. Neural Circuits *15*, 815923. https://doi.org/10.3389/fncir.2021.815923.

Ueki, Y., Karl, M.O., Sudar, S., Pollak, J., Taylor, R.J., Loeffler, K., Wilken, M.S., Reardon, S., and Reh, T.A. (2012). P53 is required for the developmental restriction in müller glial proliferation in mouse retina. Glia *60*, 1579–1589. https://doi.org/10.1002/glia.22377.

Ueki, Y., Wilken, M.S., Cox, K.E., Chipman, L., Jorstad, N., Sternhagen, K., Simic, M., Ullom, K., Nakafuku, M., and Reh, T.A. (2015). Transgenic expression of the proneural transcription factor Ascl1 in Müller glia stimulates retinal regeneration in young mice. Proc. Natl. Acad. Sci. USA *112*, 13717–13722. https://doi.org/10.1073/pnas.1510595112.

VandenBosch, L.S., Wohl, S.G., Wilken, M.S., Hooper, M., Finkbeiner, C., Cox, K., Chipman, L., and Reh, T.A. (2020). Developmental changes in the accessible chromatin, transcriptome and Ascl1-binding correlate with the loss in Müller Glial regenerative potential. Sci. Rep. *10*, 1–18. https://doi.org/10.1038/s41598-020-70334-1.

Vigouroux, R.J., César, Q., Chédotal, A., and Nguyen-Ba-Charvet, K.T. (2020). Revisiting the role of DCC in visual system development with a novel eye clearing method. Elife *9*, e51275. https://doi.org/10.7554/eLife.51275.

Viñals, F., Reiriz, J., Ambrosio, S., Bartrons, R., Rosa, J.L., and Ventura, F. (2004). BMP-2 decreases Mash1 stability by increasing Id1 expression. EMBO J. *23*, 3527–3537. https://doi.org/10.1038/sj.emboj.7600360.

Völkner, M., Wagner, F., Steinheuer, L.M., Carido, M., Kurth, T., Yazbeck, A., Schor, J., Wieneke, S., Ebner, L.J.A., del Toro Runzer, C., et al. (2022). HBEGF-TNF induce a complex outer retinal pathology with photoreceptor cell extrusion in human organoids. Nat. Commun. *13*, 6183. https://doi.org/10.1038/s41467-022-33848-y.

Völkner, M., Zschätzsch, M., Rostovskaya, M., Overall, R.W., Busskamp, V., Anastassiadis, K., and Karl, M.O. (2016). Retinal Organoids from Pluripotent Stem Cells Efficiently Recapitulate Retinogenesis. Stem Cell Rep. *6*, 525–538. https://doi.org/10.1016/j.stemcr.2016.03.001.

Wan, J., and Goldman, D. (2016). Retina regeneration in zebrafish. Curr. Opin. Genet. Dev. *40*, 41–47. https://doi.org/10.1016/j.gde.2016.05.009.

Zhong, X., Gutierrez, C., Xue, T., Hampton, C., Vergara, M.N., Cao, L.H., Peters, A., Park, T.S., Zambidis, E.T., Meyer, J.S., et al. (2014). Generation of three-dimensional retinal tissue with functional photoreceptors from human iPSCs. Nat. Commun. *5*, 4047. https://doi.org/10.1038/ncomms5047.

Stem Cell Reports, Volume *18*

Supplemental Information

ASCL1 induces neurogenesis in human Müller glia

Juliette Wohlschlegel, Connor Finkbeiner, Dawn Hoffer, Faith Kierney, Aric Prieve, Alexandria D. Murry, Alexandra K. Haugan, Isabel Ortuño-Lizarán, Fred Rieke, Sam A. Golden, and Thomas A. Reh

Figure legends

Figure S1

Markers of the presumptive fovea are already present by D57 (FWK8)

(A) Top: 2D sub-stack of a D57 fetal eye immunolabeled with OTX2 (green) and Recoverin (RCVRN, magenta). Bottom: IF whole-mount of the eye shown from the posterior. Grid boxes show the dimensions of the intact volume. Arrow points out the optic nerve; arrowhead shows the presumptive fovea. (B) D57 fetal retinal section immunolabelled with VSX2 (red), OTX2 (green), RCVRN (magenta) and DAPI (grey). Scale bar = 100 µm. Abbreviations: FWK: Fetal week; PF: presumptive fovea; ON: optic nerve; T: temporal; N: nasal.

Figure S2

SnRNA-seq and SnATAC-seq analysis of the D59 sample alone.

(A) UMAP plot of the D59 (FWK8) sample colored by cell type clusters. (A') UMAP with the different phases of the cell cycle, showing that MG are mostly in G1**.** Abbreviations: MPC: multipotent progenitor cells, Npre: neurogenic precursors cells, RGC: retinal ganglion cells, CON: cone photoreceptors, BIP: bipolar cells, MG: muller Glia, AST: astrocytes, HOR: horizontal cells, cyNpre: neurogenic precursors cells in the mitotic cycle. (B) Dot plot showing the expression of cell-type specific genes in the different cell clusters. (C) Feature plots showing the relative expression of glial markers RLBP1, SLC1A3, and NFIA. (D) Scatterplot of multipotent progenitors (MPCs) vs Muller glia (MG): genes that are significantly different in expression are colored and the top 10 differentially expressed genes are labeled. RLBP1 is significantly more highly expressed in the MG than in the MPCs, but it is not in the top 10 genes**.** (E) Scatterplot of enriched transcription factor binding motifs in peaks enriched in either MPCs or MGs as a percentage of cells. Transcription factor motifs that correspond to relative differences in RNA expression (shown in D) are labeled in panel E.

Figure S3

SnRNA-seq and SnATAC-seq analysis of the merged dataset (D59, D76C, and D76P).

(A) Dot plot showing the gene expression in the different cell clusters. (B) Stacked Bar plot showing the percentages of each cell cluster split by age (D59, D76C, and D76P). (C) Top GO biological process analysis for the MG (red) and MPC (blue) clusters for cells that are in the G1 phase of the cell cycle. (D) Scatterplot of accessible chromatin peaks that contain significantly higher abundance peaks specific to MG (red) or specific to MPC (blue).

Figure S4

PAX2+ cells in retinal organoids and retinospheres are likely astrocytes.

(A) FD150 (FWK21) fetal retinal section immunolabelled with RLBP1(red), VSX2 (green), PAX2 (cyan) and DAPI (grey). ONL: outer nuclear layer; INL: inner nuclear layer; GCL: ganglion cell layer. Scale bar = 50 µm. (B) PAX2+ are mostly absent in retinal organoids, PAX6 (red), SOX2 (green), PAX2 (cyan) and DAPI (grey). Scale bar = 100 µm. Arrowheads show PAX2+ cells. (C) MG cultures derived from RO and passaged once immunolabelled with the same markers as in (A) and (B). Scale bar = 100 µm. (D) PAX2+ cells are present in some retinospheres and (E) are more abundant in MG cultures (arrows) derived from fetal retinal tissues (retinospheres). Scale bar = 100 µm.

Figure S5

Both MG derived from human fetal retina and from retinal organoids can be reprogrammed into neurogenic precursors *in vitro.*

(A) Heatmap showing the expression the top 25 marker genes expressed in the neuronal cluster after *ASCL1* expression (from Fig 6E) averaged and normalized across the neuronal clusters of the Mutiome data (from Fig 2) and (B) plotted onto the merged UMAP of the Multiome data (from Fig 2B). (C) Pseudotime values on a UMAP plot of snRNA-seq results from MG cultures derived from retinal organoids that were infected with *ASCL1* expressing virus. (D) Stacked Bar plot showing cluster composition for the two different conditions: CTL and ASCL1. (C) and (D) are further analyzed in Figure5. (E) Pseudotime values on a UMAP plot of scRNA-seq results from MG cultures derived from fetal retina retinospheres that were infected with ASCL1 expressing virus. (F) Stacked Bar plot showing cluster composition for the two different conditions: CTL and ASCL1. (E) and (F) are further analyzed in Figure 6. (G) Integrated UMAP plot of the two different datasets (Fig 5 and Fig 6), split by conditions (G') MG cultures derived from retinospheres (Fig 6), (G'') MG cultures derived from retinal organoids (Fig 5).

Figure S6

ASCL1 overexpression combined with Notch signaling inhibition induces MG-derived OTX2+ neurons.

(A) Feature plots showing the expression values of HES1, ID1, ID3, (top panel) in the dataset obtained from retinal organoids (Fig 5), (lower panel) in the dataset obtained from fetal retina (retinospheres) (Fig 6). (B) Schematic of the experimental timeline with the Notch inhibitor. (C) Merged UMAP of the MG cultures (CTL: no virus, ASCL1 only and ASCL1 combined with Notch

inhibitor). (D) Feature plots showing the expression of neurogenic precursor maker (HES6) and neuronal markers (ELAVL3, DCX and RBFOX3). (E) Merged UMAP splits by condition; red (CTL), green (ASCL1 only) and blue (ASCL1 overexpression combined with Notch signaling inhibition). (F) Stacked Bar plots showing neurogenic and neuron cluster composition for the ASCL1 alone and the ASCL1 + Notch inhibitor conditions. (G) Feature plot showing OTX2 expression on the merged UMAP split by conditions. Arrow indicates the OTX2 neuronal cluster present only in the ASCL1+ Notch inhibition signaling condition.

Video 1

The presumptive fovea is detectable as early as D59 in the human fetal retina.

A 3D rendering of a D59 (FWK8) human fetal eye after whole mount immunostaining and clearing. Recoverin (RCVRN, magenta) labelling is only restricted to one specific region of the retina, temporal to the optic nerve. The green channel (IBA1) was used to visualize the structure retina.

Antibodies	Source (brand)	Identifier (catalogue identifier)	Dilution
Mouse anti-CRALBP (RLBP1)	abcam	ab15051	1/200
Rabbit anti-GFAP	DAKO	Z0334	1/500
Goat anti-SOX2	SC-17320	SC-17320	1/300
Rabbit anti-SOX9	Millipore	Ab5535	1/300
Sheep anti-CHX10	EXALPHA	X1179P	1/300
Mouse anti-PAX6	DSHB	Pax6-s	1/50
Rabbit anti-PAX2	Biolegend	901001	1/100
Goat anto-OTX2	R&D Systems	BAF1979	1/300
Mouse anti-TUJ1	Biolegend	802001	1/300
Rabbit anti-RCVRN	Chemicon	AB5585	1/1000
Rabbit anti-PH3	NOVUS Biologicals	NB600-1168	1/300
Mouse anti-HUCD	Life Technologies	A-21271	1/200
Goat anti-DCX	Santa Cruz	D3015	1/300
Guinea pig anti-IBA1	Synaptic Systems	234-004	1/1000
Mouse anti-Ki67	Leica	NCL-L-ki67-MM1	1/300
Mouse anti-CalBindin	Millipore	AB1778	1/300

Donkey anti-goat 568	Invitrogen	A11057	1/300
Donkey anti-goat 488	Jackson	705-545-147	1/300
Donkey anti-mouse 488	Jackson	715-605-150	1/300
Donkey anti-mouse 568	Invitrogen	A10037	1/300
Donkey anti-mouse 647	Invitrogen	A32787	1/300
Donkey anti-rabbit 488	Invitrogen	A21206	1/300
Donkey anti-rabbit 568	Invitrogen	A10042	1/300
Donkey anti-rabbit 647	Invitrogen	A31573	1/300
Donkey anti-sheep 488	Invitrogen	A11015	1/300

Table 1. List of antibodies. Columns present information about the antibodies, source, identifier, and dilution used for immunofluorescence or whole mount.

Experimental procedure

Organoid cultures

The human pluripotent stem cells line H7- BRN3-td Tomato was maintained as colonies on Matrigel coated well plates supplemented with StemFlex medium (Thermo Fisher Scientific). Medium was changed every other day. Reaching confluency, stem cells were passaged for maintenance using ReLeSR (StemCell Technologies) following manufacturer's instructions. Cells were then split, with the ratio depending on the confluency of the cells, once a week. For the differentiation protocol, we used Dispase (Gibco) (1mg/ml) to lift the colonies and let them self-aggregate into embryoid bodies (EBs) in T25 flaks with a medium containing 1/1 StemFlex and Neural induction medium (NIM, DMEMf12, 1% N2 supplement, 1% MEM non-essential amino acids, 1% Pen-Strep). Two days later, EB medium was completely switched to NIM medium. On day 6, BMP4 (R&D systems) was added to the flask and, on day 7 EBs were plated on regular cell culture treated plates with 10% FBS. Medium was changed the next day (NIM). On day 20 plated EBs were lifted using P1000 tips and were then transferred into low adhesion plates supplemented with retinal differentiation medium (RDM: DMEM/DMEMf12, 1% MEM non-essential amino acids, 2% B27, 5% FBS, 1% Pen-Strep). Retinal organoids were maintained for long term *in vitro* culture with medium changing 3 times a week on Monday, Wednesday, and Friday. Since, retinal organoids have distinct features which enable selection, non-retinal forebrain cells were manually removed. All cells (stem cells, EBs and retinal organoids) were maintained in an incubator at 37°C with 5% CO_2.

Freezing protocol

After removing the medium, plated cells were harvested using Accutase (Millipore Sigma) for 5-8 mins, 37°C. The cell suspension was then spun down at 300 RPM for 7 min at 4°C. Supernatant was removed, and cells were resuspended using a medium containing 50 % of neuronal medium, 40% FBS and 10% DMSO. Cells were then preserved in a cryotube at -80°C.

Notch inhibition in MG cultures

Four days after *ASCL1* overexpression with the lentiviral construct, we used 10 µM of PF-03084014-hydrobromide (PF4014) (Millipore Sigma) to inhibit Notch signaling *in vitro*. Medium was not changed during the three days following Notch inhibition.

Single-cell multiome sequencing data preparation and analysis

The Cellranger ARC pipeline (2.0) was used to demultiplex samples and align reads to the GRCh38 genome. First, we used 'cellranger-arc mkfastq' to convert BCL files to fastq and demultiplex samples. This was done independently for the ATAC and RNA reads. Next, cellranger arc count was used to combine ATAC and RNA reads from each cell (using cell barcodes and samples of origin), map Tn5 insertions to the genome, and align RNA reads to the genome. Data was further processed in R version 4.2.2 (R core team, 2021).

Data from cellranger arc count were loaded into Signac (1.9.0) (Stuart et al., 2021) /Seurat (4.3.0)(Hao et al., 2021). The RNA assay was processed using the default Seurat pipeline. Reads were normalized using 'NormalizeData', variable features were Identified using 'FindVariableFeatures', gene expression across cells was scaled using 'ScaleData', and PCs were created using RunPCA(). Additionally, all samples used the RNA data for clustering and UMAP. This was done using Seurat's RunUMAP() function (to create the UMAP), and the 'FindNeighbors' and 'FindClusters' functions to find clusters in the neighbor space. Clusters were identified via differential gene expression and expression of known marker genes. Cell cycle phase was approximated using Seurat's 'CellCycleScoring' function which uses a curated list of genes to determine if cells are in the S, G2M, or G2, G1 phase of the cell cycle.

The ATAC assay for each object was created using the barcoded fragment files from Cellranger arc count. Signac's 'CallPeaks' function was used to call peaks. This function calls Macs2 (2.2.7) (Zhang et al., 2008) on the fragment files to identify peaks in the sample. Cell fragment

pairs were mapped to the Macs2 called peaks using Signac's 'FeatureMatrix' function which returned a peak by cell matrix. This matrix was used for all downstream analysis. Nucleosome signal strength and TSS enrichment for each cell were calculated using Signac's 'NucleosomeSignal' and 'TSSEnrichment' functions respectively. Outliers in the QC metric categories were removed as per Signac's standard processing guidelines.

Peaks were annotated with motifs for transcription factor binding sites in the Jaspar 2020 database using Signac's 'AddMotifs' function. Signac's 'RunChromvar' function was then run to find motif accessibility z-scores across cells. The z-score values were used in the motif heatmaps and featureplots. Coverage plots were made using Signac's 'CoveragePlot' function.

Monocle 3

Pseudotime was calculated using Monocle 3. The Seurat object was loaded into Monocle3 using SeuratWrapper's 'as.cell_data_set()' function. Clusters were rerun using Monocle3's 'cluster_cells()' function and the lineage graph was created using Monocle3's 'learn_graph()' function. Monocle3's 'order_cells()' function was used to assign the pseudotime root using known markers of precursor cell types. For the D59 sample there was a small bipolar branch that was visible on UMAP but did not cluster out from the rest of the OTX2 lineage. To isolate this group of cells we used Monocle3's 'choose_graph_segments()' to select cells on this branch segment.

Cascade Heatmap plots

Cascade plots were generated as previously described (Finkbeiner et al., 2022; Todd et al., 2022). To create the heatmap, we used chromvar activity scores of the top variable motifs across the different lineages arranges over pseudotime. Similarly, RNA heatmaps were generated using the z-score (scale data) gene expression values corresponding to each of the variable motifs in the chomvar heatmap, following the same order over pseudotime.

Combining datasets

Before combining datasets, we first ran all previous computational steps on each sample independently. If the samples we were combining contained accessibility assays we merged peaks from all samples that were to be combined using BEDOPS (-m)(Neph et al., 2012) on the Macs2 peaks for each sample. Signac's 'FeatureMatrix' function was then run with the resulting merged peaks to create a new peak by cell matrix. All assays in the samples that were to be

combined were then downsampled to similar average read depth using R and DropletUtils 'downsampleMatrix' function. If the samples were sequenced at the same time samples were merged using Signac/Seurat's 'merge' function, and standard Seurat/Signac normalization, dimensional reduction, clustering, and visualization was performed as described above. For integrated samples (Reprogramming 2 and integrated reprogramming 2 and 4) the objects were split by samples then integrated with Seurat's 'IntegrateData' function using anchors calculated using Seurat's 'FindIntegrationAnchors' function. Then normalization, dimensional reduction, and visualization steps were repeated as previously described.

Peak-gene and TF-gene linkage

For Multiome, samples genes were linked to peaks using Signac's 'LinkPeaks' function using the default parameters. To investigate whether targets of a specific transcription factor were up or downregulated between groups, differential gene expression was performed between the groups of interest and differentially expressed genes were aggregated by group. For each differentially expressed gene linked peaks with a positive score were selected giving us peaks linked to differentially expressed genes for each population. Percentage of genes with TF binding motifs in linked peaks for DE genes for each population were calculated by inputting the linked peaks from each population into Signac's 'FindMotifs' function. The resulting percentages were plotted against each other as a scatterplot colored according to the average $\log_2$ fold change from Seurat's 'FindMarkers()' function.

References

Finkbeiner, C., Ortuño-Lizarán, I., Sridhar, A., Hooper, M., Petter, S., & Reh, T. A. (2022). Single-cell ATAC-seq of fetal human retina and stem-cell-derived retinal organoids shows changing chromatin landscapes during cell fate acquisition. *Cell Reports*, *38*(4). https://doi.org/10.1016/j.celrep.2021.110294

Hao, Y., Hao, S., Andersen-Nissen, E., Mauck, W. M., Zheng, S., Butler, A., Lee, M. J., Wilk, A. J., Darby, C., Zager, M., Hoffman, P., Stoeckius, M., Papalexi, E., Mimitou, E. P., Jain, J., Srivastava, A., Stuart, T., Fleming, L. M., Yeung, B., … Satija, R. (2021). Integrated analysis of multimodal single-cell data. *Cell*, *184*(13), 3573-3587.e29. https://doi.org/10.1016/j.cell.2021.04.048

Neph, S., Kuehn, M. S., Reynolds, A. P., Haugen, E., Thurman, R. E., Johnson, A. K., Rynes, E., Maurano, M. T., Vierstra, J., Thomas, S., Sandstrom, R., Humbert, R., & Stamatoyannopoulos, J. A. (2012). BEDOPS: High-performance genomic feature operations. *Bioinformatics*, *28*(14), 1919–1920. https://doi.org/10.1093/bioinformatics/bts277

Stuart, T., Srivastava, A., Madad, S., Lareau, C. A., & Satija, R. (2021). Single-cell chromatin state analysis with Signac. *Nature Methods*, *18*(11), 1333–1341. https://doi.org/10.1038/s41592-021-01282-5

Todd, L., Jenkins, W., Finkbeiner, C., Hooper, M. J., Donaldson, P. C., Pavlou, M., Wohlschlegel, J., Ingram, N., Rieke, F., & Reh, T. A. (2022). Reprogramming Müller glia to regenerate ganglion-like cells in adult mouse retina with developmental transcription factors. *SCIENCE ADVANCES*. https://doi.org/10.1126/sciadv.abq7219

Zhang, Y., Liu, T., Meyer, C. A., Eeckhoute, J., Johnson, D. S., Bernstein, B. E., Nussbaum, C., Myers, R. M., Brown, M., Li, W., & Shirley, X. S. (2008). Model-based analysis of ChIP-Seq (MACS). *Genome Biology*, *9*(9). https://doi.org/10.1186/gb-2008-9-9-r137
